# Gender–specific Single Transcript Level Atlas of Vasopressin and its Receptor (AVPR1a) in the Mouse Brain

**DOI:** 10.1101/2024.12.09.627541

**Published:** 2024-12-10

**Authors:** Anisa Gumerova, Georgii Pevnev, Funda Korkmaz, Uliana Cheliadinova, Guzel Burganova, Darya Vasilyeva, Liam Cullen, Orly Barak, Farhath Sultana, Weibin Zhou, Steven Sims, Victoria Laurencin, Tal Frolinger, Se-Min Kim, Ki A. Goosens, Tony Yuen, Mone Zaidi, Vitaly Ryu

**Affiliations:** Center for Translational Medicine and Pharmacology, Icahn School of Medicine at Mount Sinai, New York, NY 10029

## Abstract

Vasopressin (AVP), a nonapeptide synthesized predominantly by magnocellular hypothalamic neurons, is conveyed to the posterior pituitary *via* the pituitary stalk, where AVP is secreted into the circulation. Known to regulate blood pressure and water homeostasis, it also modulates diverse social behaviors, such as pair–bonding, social recognition and cognition in mammals including humans. Importantly, AVP modulates social behaviors in a gender–specific manner, perhaps, due to gender differences in the distribution in the brain of AVP and its main receptor AVPR1a. There is a *corpus* of integrative studies for the expression of AVP and AVPR1a in various brain regions, and their functions in modulating central and peripheral actions. In order to purposefully address sexually dimorphic and novel roles of AVP on central and peripheral functions through its AVPR1a, we utilized RNAscope to map *Avp* and *Avpr1a* single transcript expression in the mouse brain. As the most comprehensive atlas of AVP and AVPR1a in the mouse brain, this compendium highlights the importance of newly identified AVP/AVPR1a neuronal nodes that may stimulate further functional studies.

## INTRODUCTION

Vasopressin (AVP), a nonapeptide synthesized primarily by magnocellular neurons of the paraventricular nucleus (PVH) and supraoptic nucleus (SO) of the hypothalamus, is conveyed along axons of magnocellular neurons *via* the pituitary stalk to the posterior pituitary, where AVP is released into circulation to exert hormonal functions (1, 2). Involved in the regulation of blood pressure and water balance (3, 4), AVP also regulates diverse social behaviors, such as pair–bonding, social recognition and cognition in all mammals, including humans (5–9). It should be noted that similar to OXT, AVP is evolutionarily conserved across invertebrates and vertebrate taxa, differing from OXT by just only two amino acids. Importantly, AVP modulates social behaviors in gender–specific manner, perhaps due to gender differences in AVP and its receptor expression in the brain.

AVP receptors are G protein–coupled receptors consisting of two major subtypes, the V1 receptor (AVPR1) and V2 receptor (AVPR2) (10). AVPR1 has two subtypes, namely AVPR1a and AVPR1b that mediate the effects of AVP on social behaviors. Avpr1a will be the overarching focus of this study given its abundance and ubiquity in brain regions (9). In fact, differences in AVPR1a genetic variability and expression patterns determine specific social phenotypes (11–15). There is evidence that AVPR1a is involved in maternal care, pair–bonding, behavioral aggression, anxiety, social recognition and social play (6, 9, 13, 16–19). The gender–dependent distribution of Avpr1a across the brain in different species provides a proxy for the distribution of AVP binding, and therefore, provides further evidence for central AVP neural nodes of physiologic relevance.

Blocking AVPR1a inhibits social recognition in the rat, while AVPR1a knockout mice fail to display social recognition (16, 20, 21). These effects of AVP in enhancing social recognition is mediated *via* AVPR1a in the lateral septum (20, 21). Gender differences in Avpr1a binding densities have been described in several brain sites of Wistar rats. Namely, males display higher AVPR1a binding densities in the following forebrain areas: somatosensory and piriform cortex, medial posterior bed nucleus of the stria terminalis (BNST), nucleus of the lateral olfactory tract (LOT), anteroventral thalamic nucleus (VA), tuberal lateral hypothalamus (LH), stigmoid hypothalamus (Stg), and dentate gyrus (DG) (10). In male prairie voles, central AVP infusion facilitates selective aggression associated with pair bond formation and partner preferences, and the Avpr1a antagonist 1-(*β*-mercapto-*β, β*-cyclopentamethylene propionic acid) does not seem to inhibit aggression (22). In contrast, central AVP infusion induces a partner preference in female prairie voles (23). Comparative analysis of AVPR1a distribution has revealed higher densities of AVPR1a binding in the ventral pallidum, amygdala, and thalamus of prairie voles than that of meadow or montane voles (24, 25). It has also been reported that AVPR1a antagonism specifically in the ventral pallidum prevents mating–induced partner preferences in male prairie voles (26).

Although osmotically stimulated AVP release with short latency and duration from hypothalamic PVH and SO magnocellular axon terminals occurs in the pituitary (27, 28), it is also released locally from somata and dendrites in the SO with a longer delay responding to osmotic challenge (29, 30). Such local release is likely to facilitate autocrine and/or paracrine regulation of SO–magnocellular neuronal activity and inhibit further systemic AVP output (30, 31). Of note, somatodendritic AVP release in response to direct hypertonic stimulation is attenuated by V1/V2 receptor antagonism, implying that AVP may facilitate its own release by acting on autoreceptors within magnocellular neurons of the SO (32). The importance of autofacilitation to local AVP release may lay in fine–tuned regulation of AVP actions towards physiologic demands. Alternatively, AVP could putatively diffuse over longer distances to bind to adjacent receptors. The relative contribution of AVP autoreceptor subtypes, including AVPR1a, to this phenomenon awaits further clarification.

Current research is evolving to consider anterior and posterior pituitary hormones, traditionally thought of as master regulators of a single physiological target, as affecting multiple bodily systems, either directly or *via* their receptors in the brain (33, 34). Non–traditional actions of AVP include its ability to affect skeletal homeostasis, wherein it negatively regulates osteoblasts and stimulates osteoclasts. This explains the bone loss that accompanies low blood sodium levels in patients with high AVP levels (35). Furthermore, we have shown that AVP (via AVPR1a) and oxytocin (via OXTR) have opposing skeletal actions—effects that may relate to the pathogenesis of bone loss in chronic hyponatremia, and pregnancy and lactation, respectively (35–38).

Detecting specific AVPR1a and AVPR1b in the brain has had limitations for a long time due to the availability of only nonselective radioligands, such as tritium labeled (^3^H) AVP ligands, which bind to both receptors (39). Although there is a large body of integrative studies for the expression of AVP and AVPR1a in various brain regions, and their functions in regulating central and peripheral actions, there remains the need for detailed, gender–specific mapping of the AVP/AVPR1a neuronal nodes in the brain. We utilized RNAscope—a technology that detects single RNA transcripts—to create a comprehensive atlas of AVP and AVPR1a in the mouse brain. It may seem somewhat remarkable that newly discovered brain areas for receptors of such evolutionarily conserved and well–studied hormones AVP and OXT are currently emerging, with inferences to novel functions. We believe that this atlas of AVP and its AVPR1a in concrete brain sites at a single transcript level should provide a resource to neuroscientists to deepen our understanding of classical and novel central and peripheral functions of AVP by interrogating AVPR1a site–specifically.

## RESULTS

AVP receptors are G protein-coupled receptors consisting of two major subtypes, the AVPR1 and AVPR2 (10). In turn, AVPR1 is divided into AVPR1a and AVPR1b receptor subtypes that mediate the effects of AVP in the brain on social behaviors. In this study, we have provided not only a distribution mapping of AVP and AVPR1a in the brain, but also assessed gender differences in their expression by RNAscope. Allowing the detection of single transcripts, RNAscope uses ~20 pairs of transcript–specific double *Z*–probes to hybridize 10–μm–thick whole brain sections. Preamplifiers first hybridize to the ~28–bp binding site formed by each double *Z*–probe; amplifiers then bind to the multiple binding sites on each preamplifier; and finally, labeled probes containing a chromogenic enzyme bind to multiple sites of each amplifier.

RNAscope data were quantified on sections from coded 3 female and 3 male mice. For simplicity and clarity in the graphs, a scatter plot has been shown for 3 nuclei, sub–nuclei and regions with the highest AVP and AVPR1a transcript densities as well as their absolute transcript counts. Each section was viewed and analyzed using CaseViewer 2.4 (3DHISTECH, Budapest, Hungary) and QuPath v.0.2.3 (University of Edinburgh, UK). The *Atlas for the Mouse Brain in Stereotaxic Coordinates* (40) was used to identify every nucleus, sub–nucleus or region, which was followed by manual counting of *Avp* and *Avpr1a* transcripts by two independent observers (V.R. and A.G.) in every tenth section using a tag feature. Receptor density was calculated by dividing the absolute receptor number by the total area (μm^2^, ImageJ) of every nucleus, sub–nucleus or region. The highest *Avp* and *Avpr1a* values in the brain regions are presented as means ± SE and compared with those of the opposite gender. Photomicrographs were prepared using Photoshop CS5 (Adobe Systems) only to adjust brightness, contrast and sharpness, to remove artifacts (*i.e.*, obscuring bubbles), and to make composite plates.

**Figure 1:**
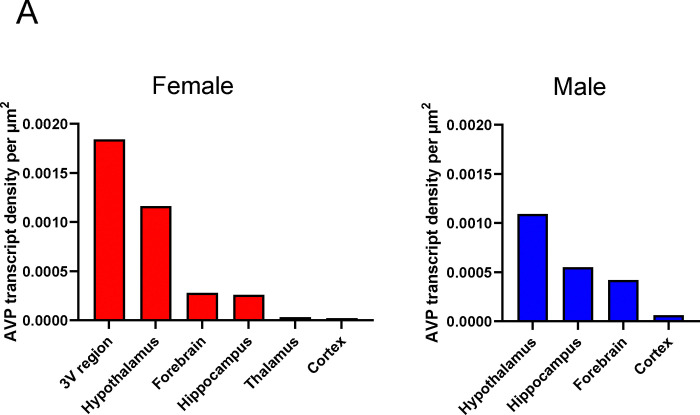

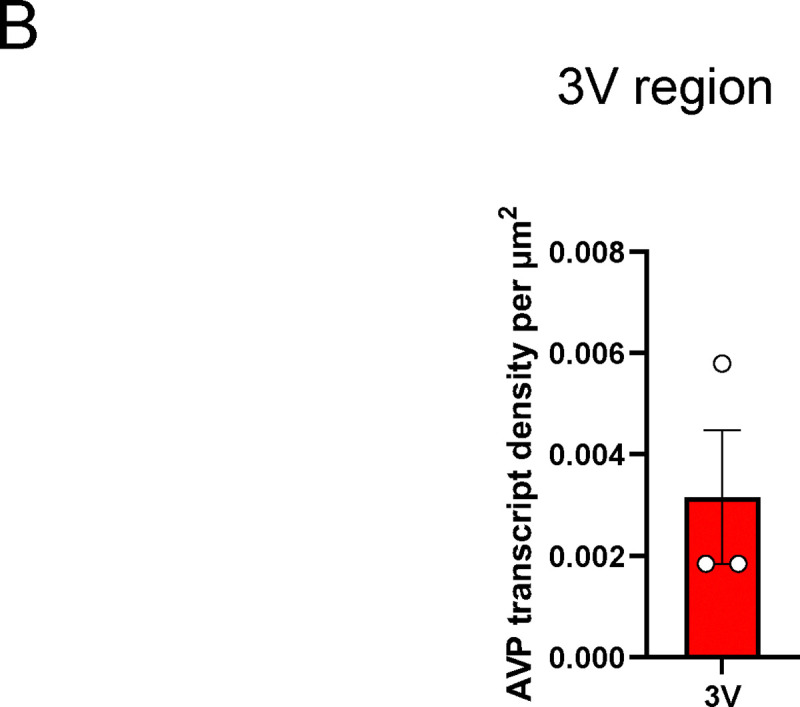

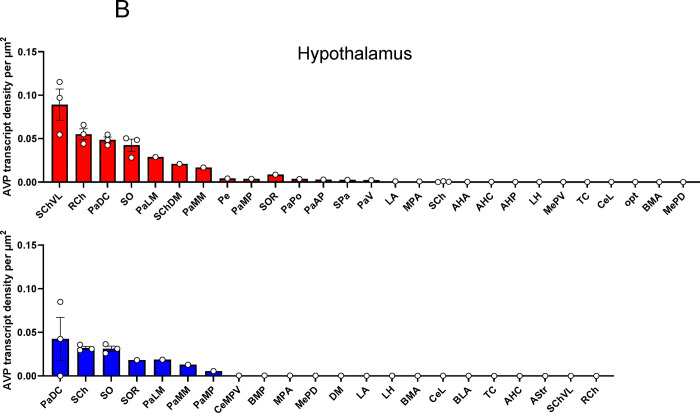

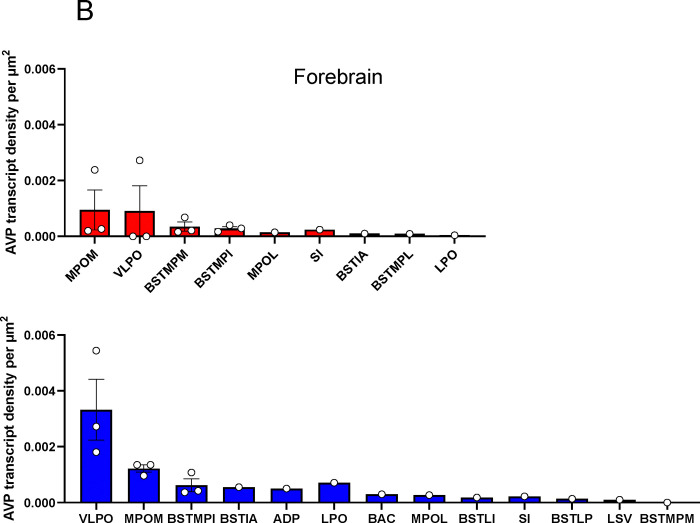

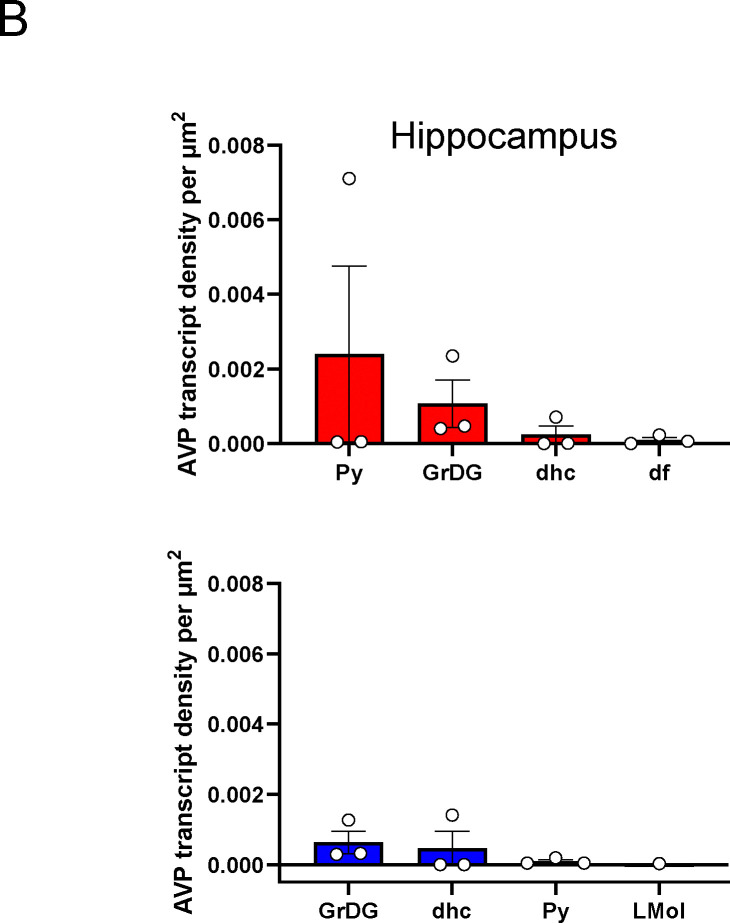

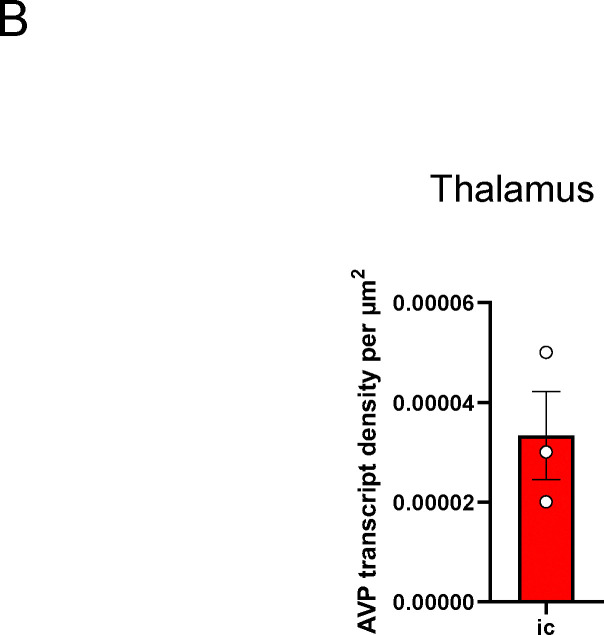

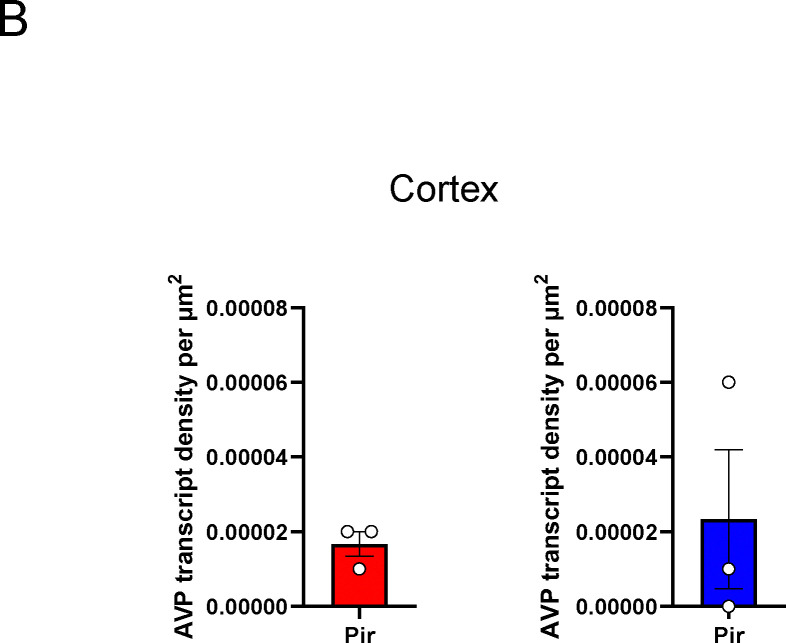

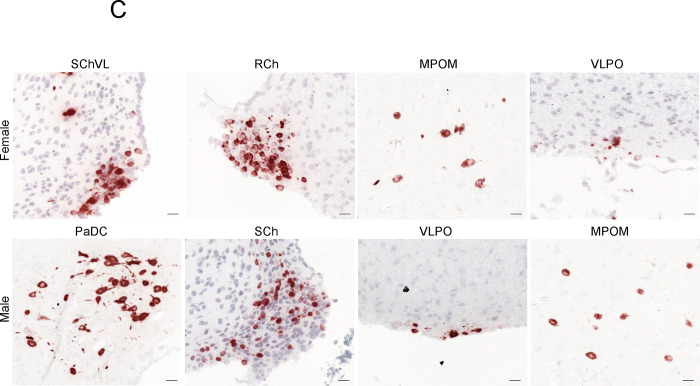
Gender–specific *Avp* transcript density in the brain. (**A**) *Avp* transcript density in main brain regions detected by RNAscope. Female 3V region and hypothalamus and male hypothalamus had the highest *Avp* transcript density. (**B**) Representative micrographs of some of the hypothalamic and forebrain regions with highest *Avp* expression. Scale bar: 20 μm. (**C**) *Avp* transcript density in nuclei, sub–nuclei and regions of the hypothalamus, forebrain, hippocampus and cerebral cortex. Note, that *Avp* transcripts were found only in the female 3V ependymal layer and thalamus.

RNAscope revealed *Avp* expression in the hypothalamus, forebrain, hippocampus and cortex of both female and male mice (Fig. 1A), however, *Avp* expression in the 3^rd^ ventricular region and thalamus was found only in the female mouse (Fig. 1B). Whereas the numbers of *Avp*–expressing cells were greater in females compared with males in the hypothalamus (607 vs. 471), 3^rd^ ventricular region (7 vs. 0) and thalamus (2 vs. 0), those numbers were lower in the hippocampus (58 vs. 118), forebrain (50 vs. 77) and cortex (5 vs. 6) [see Appendix 1 for Glossary and Supplementary Fig. 1 for raw count graphs].

The highest *Avp* transcript densities were detected in following brain nuclei, sub–nuclei and regions of female and male mice, respectively: ventricular region—3V only for females, hypothalamus—SChVL and PaDC, forebrain—MPOM and VLPO, hippocampus—Py and GrDG, thalamus—ic only for females and cerebral cortex—Pir for both (Fig. 1B). Representative micrographs of some of the hypothalamic and forebrain regions with highest *Avp* expression are shown in Fig. 1C.

**Figure 2:**
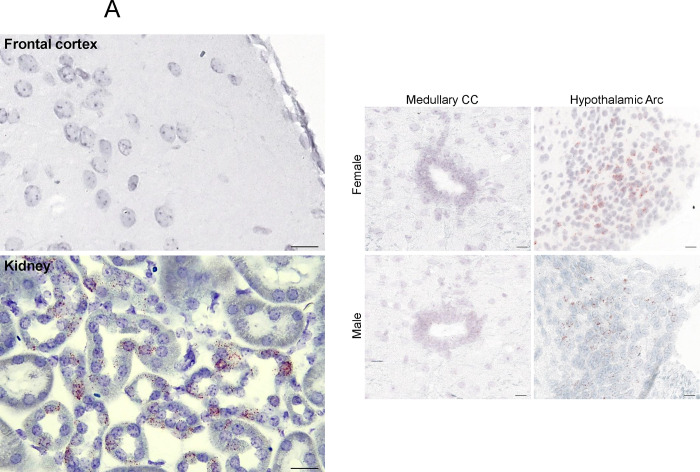

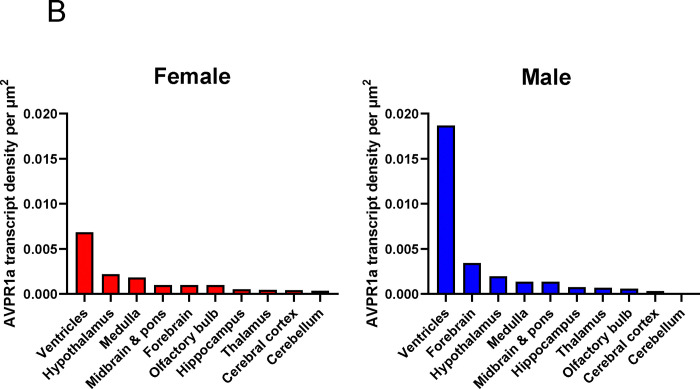

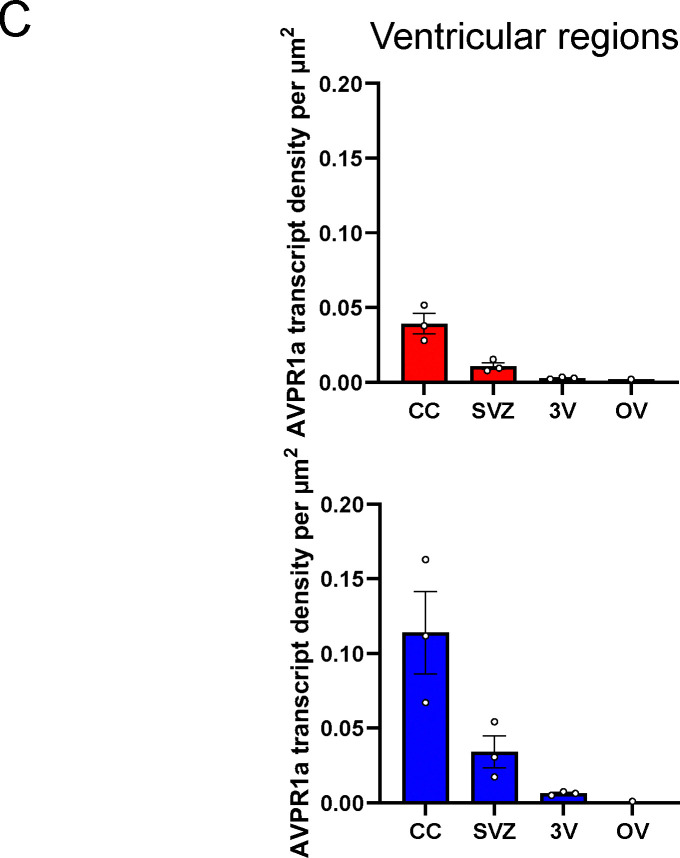

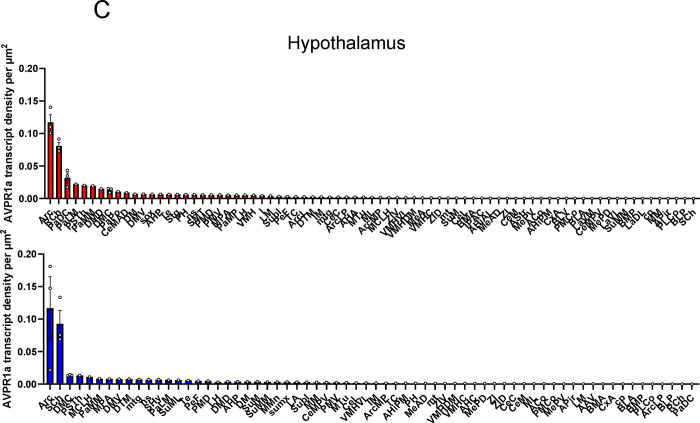

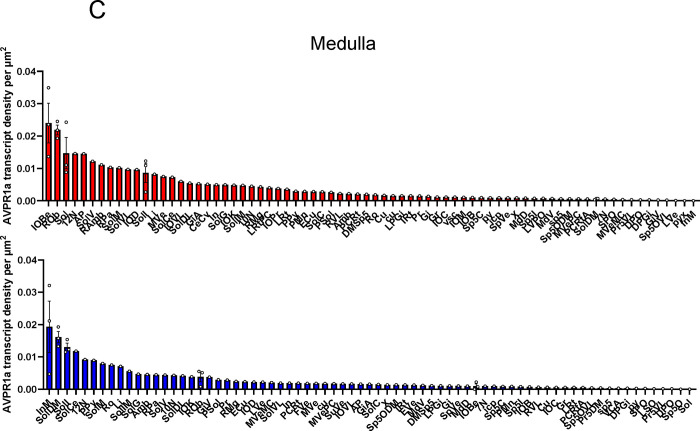

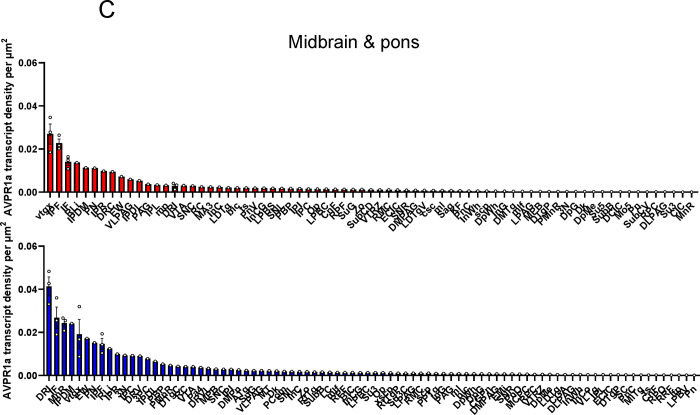

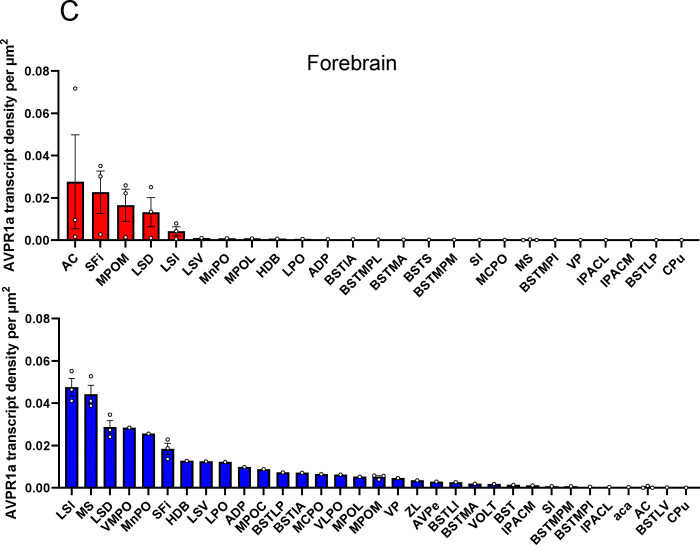

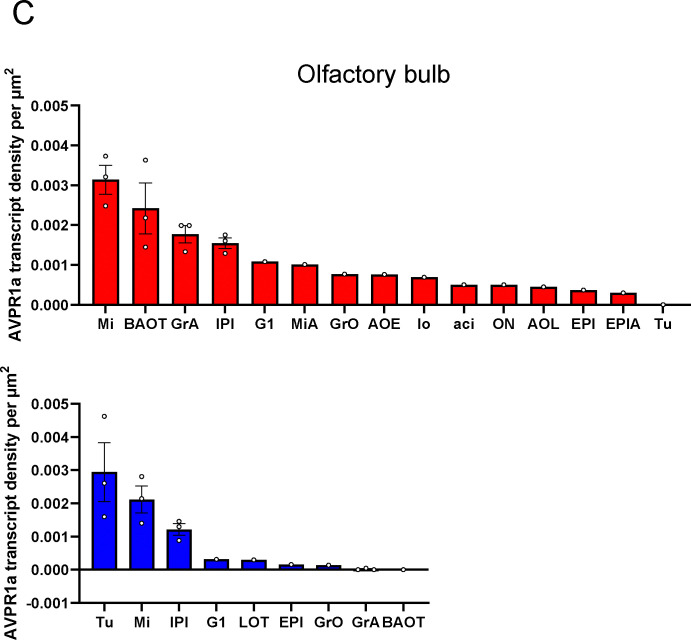

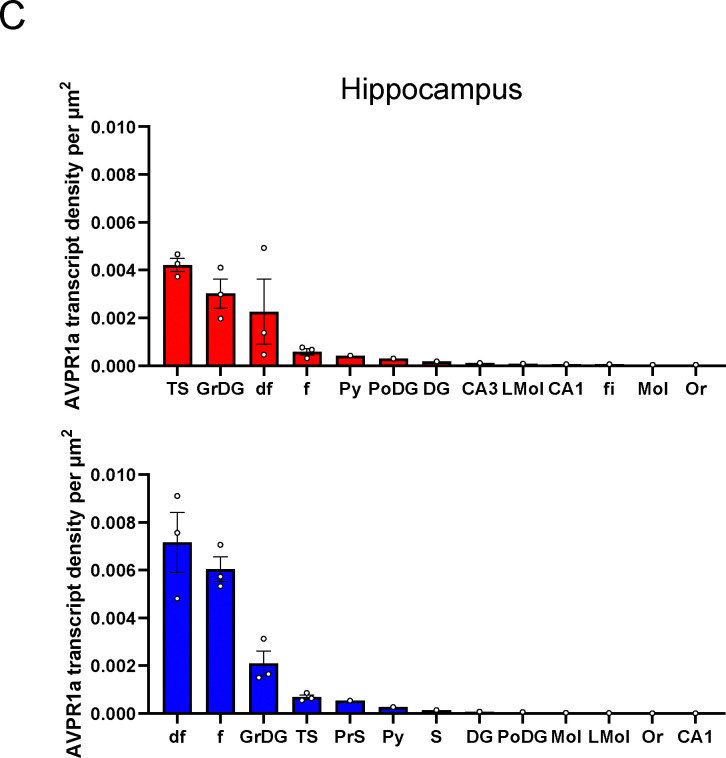

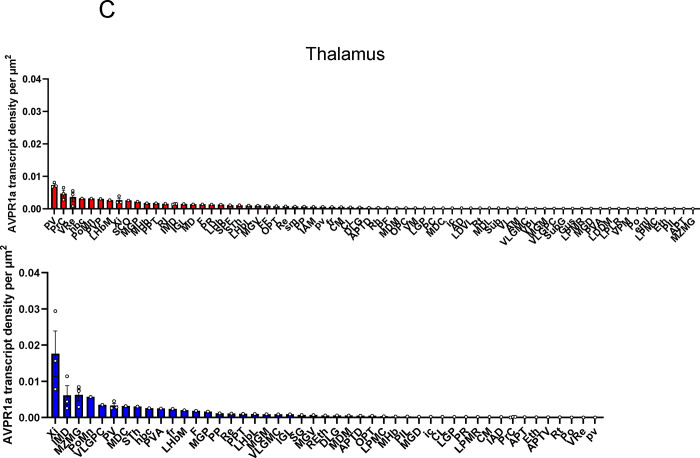

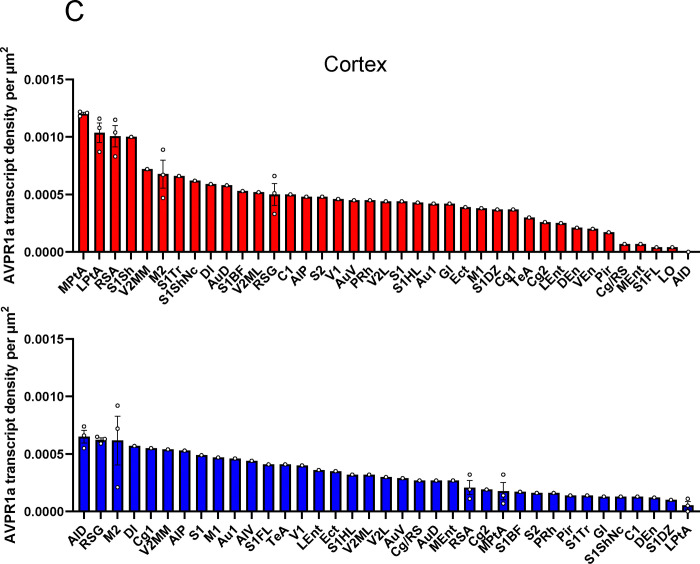

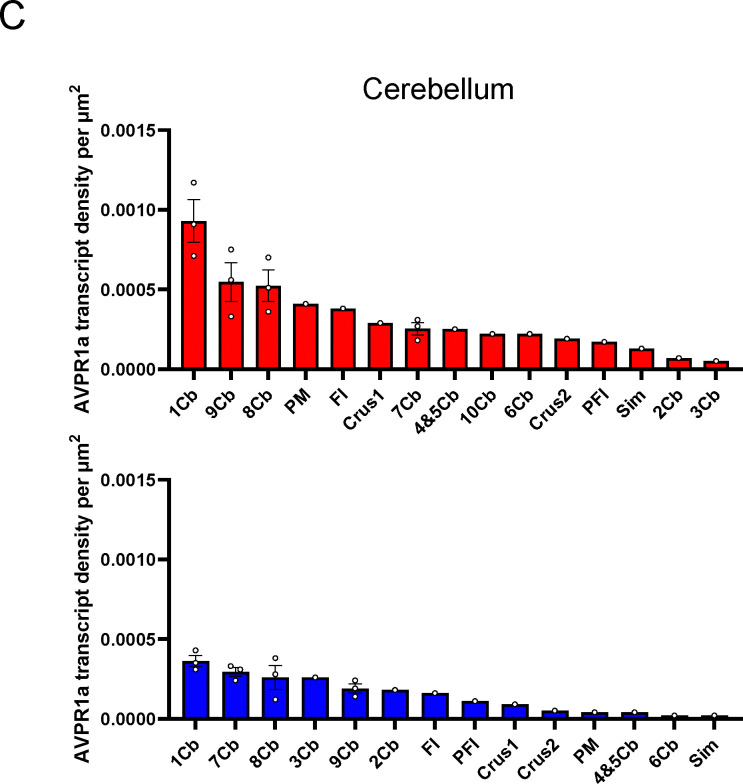
Gender–specific *Avpr1a* transcript density in the brain. (**A**) Probe specificity was established by a positive signal in renal tubules of the kidney (positive control) with an absent signal in the frontal association cortex (FrA) (negative control). Also shown are representative micrographs of medullary central canal (CC) and hypothalamic arcuate nucleus (Arc). (**B**) *Avpr1a* transcript density in main brain regions detected by RNAscope. (**C**) *Avpr1a* transcript density in nuclei, sub–nuclei and regions of the ventricular regions, hypothalamus, medulla, midbrain and pons, forebrain, olfactory bulb, hippocampus, thalamus, cortex and cerebellum. Scale bar: 25 μm (controls) and 20 μm in (A).

We report the expression of the *Avpr1a* in 398 and 375 brain nuclei, sub–nuclei and regions of the female and male mice, respectively. *Avpr1a* transcripts were detected bilaterally, with no apparent ipsilateral domination. Probe specificity was established by a positive signal in renal tubules of the kidney (positive control) with an absent signal in the FrA of the frontal cortex (negative control) (Fig. 2A). Representative micrographs of gender–specific medullary CC and hypothalamic Arc with highest *Avpr1a* expression also are shown in Fig. 2A.

In the female, total *Avpr1a* transcript numbers were the highest in the medulla followed, in descending order, by the hypothalamus, cortex, midbrain and pons, forebrain, thalamus, cerebellum, hippocampus, olfactory bulb and ventricular regions (Supplementary Fig. 2A). In the male, *Avpr1a* transcripts were the highest in the forebrain, followed by the medulla, midbrain and pons, hypothalamus, cortex, hippocampus, thalamus, olfactory bulb, cerebellum and ventricular regions (Supplementary Fig. 2).

Using the RNAscope dataset we further calculated *Avpr1a* density in all brain divisions (Fig. 2B), nuclei, sub–nuclei and regions (Fig. 2C). Highest *Avpr1a* densities in the female and male mice, respectively, were noted in nuclei, sub–nuclei and regions as follows: ventricular regions—CC ependymal region for both with 2.92–fold greater in the male; hypothalamus—Arc for both; medulla—IOBe and InM; midbrain and pons—vtgx and DRI; forebrain—AC and LSI; olfactory bulb—Mi and Tu; hippocampus—TS and df; thalamus—PV and Xi; cortex—MPtA and AID and cerebellum—1Cb for both with 2.58-fold greater in the female (Fig. 2C).

**Figure 3:**
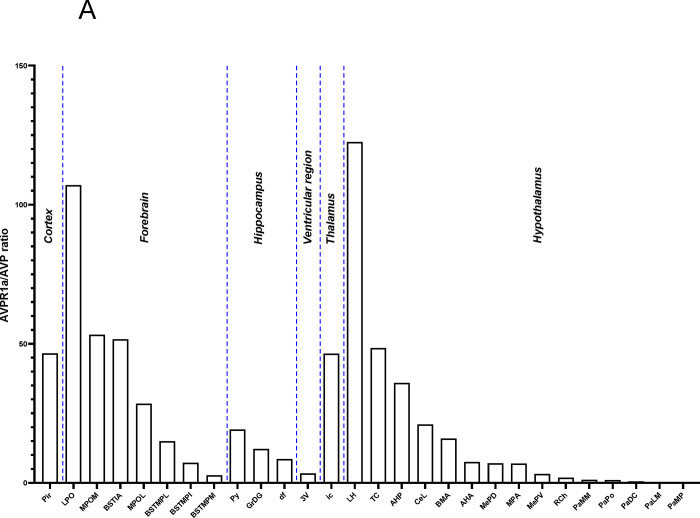

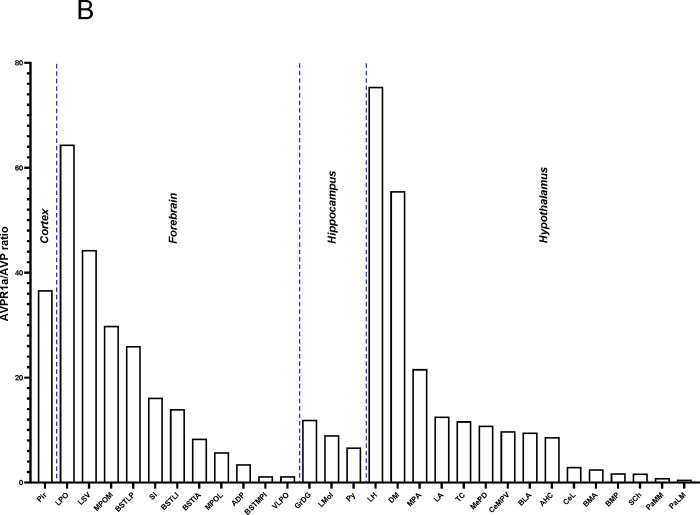
*Avp* and *Avpr1a* co-localization in the brain. We found that various nuclei and sub–nuclei exhibited *Avp* and *Avpr1a* co-localization in the brain of both sexes. (**A**) Female and (**B**) male *Avpr1a* to *Avp* ratios in the cortex, forebrain, hippocampus, 3^rd^ ventricular region, thalamus and hypothalamus.

**Figure 4:**
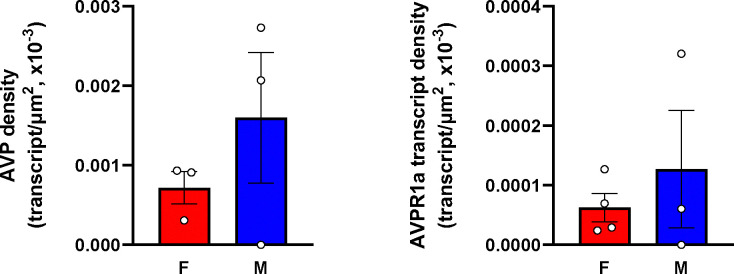
*Avp* and *Avpr1a* transcript densities in the pituitary gland. RNAscope revealed *Avp* and *Avpr1a* expression in the posterior pituitary lobe with *Avp* and *Avpr1a* transcript densities that were higher in male compared with female mice.

In addition, RNAscope analysis revealed that various brain nuclei, sub–nuclei and regions in both female and male mice co–localized *Avp* and *Avpr1a* transcripts. *Avpr1a* to *Avp* ratios within the same brain nucleus, sub–nucleus and region in both sexes are demonstrated in Figs. 3A, B. Finally, RNAscope showed *Avp* and *Avpr1a* expression in the posterior pituitary lobe with *Avp* and *Avpr1a* transcript densities that were higher in male compared with female mice, however, without statistical significance (Fig. 4).

## DISCUSSION

Here, we attempted to integrate previous information on gender–specific AVP and its AVPR1a expression in the murine brain. AVPR1a is the most abundant and wide spread receptor in the brain (9) that plays a dominant role in regulating behavior. In addition, we focused towards paradigm–shifting non–traditional roles of central AVP signaling in light of newly discovered AVPR1a. We report AVP expression in 41 female and 13 male brain nuclei, sub–nuclei and regions. Moreover, we identified abundant AVPR1a expression in 398 female and 375 male brain nuclei, sub–nuclei and regions. Therefore, this report is the most exhaustive atlas of brain *Avp* and *Avpr1a* expression at the single transcript level.

It has been reported that AVP synthesis and AVP fiber projections are sexually dimorphic in specific brain sites [for review, see: (10)]. To our knowledge, the first discovery of the sexually dimorphic nature of AVP in the rat brain was made by De Vries *et al*. (41). That is, males displayed more AVP–immunoreactive fibers in the lateral septum and lateral habenular nucleus over females (41). Surprisingly, gender differences in AVP fiber density in the LS and medial amygdala (MeA) originate from the BNST, given only lesions to the BNST, but not the PVH, result in decreased AVP fiber density in the LS (42, 43). In adult rats, AVP fiber density from the BNST and MeA are dependent on circulating gonadal hormones, as gonadectomy eliminates AVP expression and hormone replacement restores AVP fiber network (44, 45). Nonetheless, gonadal hormones appear to only partially explain gender difference in AVP expression because both females and males, exposed to a similar gonadal steroid hormone regime, still differ sexually (46, 47).

Magnocellular neurons of the PVN, SO and SCh of the hypothalamus are the predominant source of AVP synthesis. Hypothalamic AVP synthesis of most rodent species is similar between males and females in the PVH and SO of mice (48, 49); PVH, SO and SCh of voles (50, 51); PVH, MPOA, LH and AHA of Mongolian gerbils and Chinese striped hamsters (52) [for review, see: (10)]. In concordance with these reports, we also find no gender difference in *Avp* synthesis, as evidenced by similar numbers of *Avp*–expressing neurons, in the PVH and SO of mice; however, we did note gender differences in *Avp* expression density in specific hypothalamic nuclei. Notably, *Avp* expression density was higher in several PVH (PaLM and PaMM) and suprachiasmatic (SChVL and RCh) subnuclei of female compared to male mice. No gender differences in SO–*Avp* expression density were found.

Furthermore, *Avpr1a* transcript density was highest in the arcuate nucleus (Arc) and retrochiasmatic sub–nucleus (RCh) in female compared with Arc and suprachiasmatic nucleus (SCh) of male mice. *Avpr1a* expression in the Arc has previously been reported (53), however, its role was unclear until a recent report demonstrating a critical involvement of Arc–NPY in the regulation of fluid homeostasis and the induction of salt water–induced hypertension through AVP modulation in the SO (54); the latter receives direct projections from the Arc (55). In contrast, it is plausible, but, by no means proven, that PVH– and/or SO–AVP may modulate Arc anorexigenic neurons to inhibit ingestive behavior, which has previously been shown with PVH oxytocinergic neurons (56). Indeed, increasing evidence suggests that AVP reduces feeding in mammals (57, 58).

It has been reported that in the rat, AVP is an important output of the SCh targeting AVP cells in other hypothalamic areas—its release into the CSF peaks in the early morning and declines later in a day (59). Specifically, SCh–AVP secreted during late sleep activates osmosensory afferents to AVP neurons in the SO and organum vasculosum of the lamina terminalis (60, 61). Similar to rodents, studies in humans also determined that the main AVP projections from the SCh target the anteroventral hypothalamic area, sub–PVH as well as ventral parts of the PVH and DMH––a remarkable evolutionary conservation of SCh innervation from rodent to human (62, 63). The fact that the SCh is another brain nucleus with high AVP and AVPR1a expression density (greater in males vs. females), accentuates an important role of SCh–AVP in circadian rhythmicity, notably impacting neuroendocrine day/night rhythms, feeding timing, period, precision, and synchronization of SCh neurons (59, 64, 65).

In the hindbrain, the highest *Avpr1a* transcript density was noted in the inferior olive, beta subnucleus (IOBe) of female and intermedius nucleus (InM) of male mice. It has been reported that AVP fibers are apparent in the hindbrain, such as the parabrachial nucleus, locus coeruleus, and near inferior olive nuclei (66). In this regard, *Avpr1a* mRNA expression has been noted in the inferior olive (53). Given this nucleus has been implicated in various functions including learning and timing of movements, it is possible that AVPR1a in the inferior olive may be activated by the paracrine release of AVP from distant nuclei, such as the SCh, to control motor learning and timing. Alternatively, AVPR1a in the inferior olive may respond to other ligands (for example, OXT) found in nearby regions (67). The role of AVPR1 in the InM of male mice is less clear, but because the InM sends monosynaptic projections to the NTS (68) that is essential for blood pressure control by AVP and receiving information from the cardiovascular receptors (69), a possible coordinated control by hindbrain AVP of blood pressure and cardiovascular function.

Although the midbrain, pons and forebrain displayed less abundant *Avpr1a* transcript density, they revealed further gender differences. In the midbrain and pons, the highest *Avpr1a* density was in the ventral tegmental decussation (vtgx) in females and dorsal raphe nucleus, interfascicular part (DRI) in males. AVP and OXT in the ventral tegmental area are known to regulate social interactions with rewarding properties. Indeed, humans, as inherently social beings, show a strong inclination to affiliate and share their emotions with each other (70, 71). Gender differences in ventral tegmental AVPR1a make biological sense, as social interaction of females, specifically, with pups and, generally, with counterparts throughout their lives, have rewarding properties fundamental to maternal behavior and survival. Modulation of AVPR1a in the dorsal raphe nucleus has also been linked to social and emotional behaviors (10, 72, 73). Sexual dimorphism in AVP innervation of and AVPR1a expression in the DRI appears to imprint dimorphic social behaviors. That is, AVPR1a blockade in the lateral habenular nucleus (LHb) of males, but not females who have lesser AVP innervation of the LHb and dorsal raphe nucleus, results in reduced urine marking to unfamiliar males and ultrasonic vocalizations to unfamiliar, sexually receptive females, whereas AVPR1a blockade in the dorsal raphe nucleus of only males reduces urine marking to unfamiliar males (72).

In the forebrain, both sexes displayed high *Avpr1a* transcript density in septal nuclei. The highest *Avpr1a* density was in the anterior commissural nucleus (AC) within the septal nuclei of females, and lateral (LSI) and medial (MS) septal nuclei of males. It is not surprising that *Avpr1a* transcript density was significantly greater in septal nuclei of males than females given in many rodent species males have more AVP–immunoreactive fibers in the lateral septum (41). Notably, the effects of AVP on social recognition is mediated *via* AVPR1a in the lateral septum (20, 21). For example, AVPR1a blockade inhibits social recognition in the rat, while AVPR1a knockout mice fail to display social recognition (16, 20, 21).

Despite *Avpr1a* transcript densities in other brain divisions were significantly lower, sexually dimorphic differences are worth mentioning here. In the olfactory bulb, females had the high *Avpr1a* density in the mitral cell layer (Mi), whereas males had high receptor expression in the olfactory tubercle (Tu). A population of AVP neurons in the olfactory bulb of the rat that play a role in social recognition via AVPR1a has been reported (74). Silencing the AVPR1a by siRNA impairs habituation/dishabituation to juvenile cues, but not to volatile odors (74). Of note, AVP is a retrograde signal that filters activation of the Mi cells in the ewe, likely through presynaptic modulation of norepinephrine or acetylcholine. The secretion of both transmitters is stimulated by AVP in the olfactory bulb (74, 75). The functional relevance of AVP signaling *via* AVPR1a activation in the Tu requires additional studies. There is, however, evidence in the rat that AVP *via* AVPR1a has, at least, an indirect impact on Tu function, as seen by a reduction in activation responding to a noxious odor of butyric acid, when the AVPR1a is blocked (76). The presence of AVPR1a in the hippocampus, thalamus, cortex, and ventricular regions are consistent with the reported effects of AVP on memory (77), emotional and reward–motivated behavior (78), blood pressure (79), blood flow and CSF production (80). Functional roles of many other nuclei shown here to express AVPR1a and not mentioned in this report are much less clear. The importance of revealing novel AVP–triggered functions by interrogating AVPR1a site–specifically, will require further investigations.

Collectively, our results provide compelling evidence of distinct and novel AVP/AVPR1a neuronal nodes in the brain. While studies on central AVP signaling and its control of blood pressure, water balance and diverse social behaviors in mammals, occupy the vast majority of the literature (3–9), we expect that this comprehensive compendium of gender–specific AVP/AVPR1a expression in the brain will deepen our understanding of the functional and neuroanatomical basis underlying old and new paradigm–shifting functions of central AVP signaling. As appears to be the case for most brain areas, the original discovery of function tends to become dogma thereby leading to an oversimplification of multiple functions of those brain areas as they interact in circuits. This contrasts the rudimentary attribution of a single function *per* brain area. Finally, the approach of direct mapping of receptor expression in the brain and periphery provides the groundwork for greater discernment of new functional arrangements of ancient pituitary glycoprotein hormones and nonapeptides, such as AVP and OXT, and provide helpful pointers towards improving pharmacological interventions in disease.

## METHODS

### Mice

Adult mice (~3 to 4–month–old) were housed in a 12 h:12 h light : dark cycle at 22 ± 2 °C with *ad libitum* access to water and regular chow. All procedures were approved by the Mount Sinai Institutional Animal Care and Use Committee and are in accordance with Public Health Service and United States Department of Agriculture guidelines.

### RNAscope

For RNAscope, mice were anesthetized with isoflurane (2–3% in oxygen; Baxter Healthcare, Deerfield, IL) and transcardially perfused with 0.9% heparinized saline followed by 10% Neutral Buffered Formalin (NBF). Brains were promptly extracted and post-fixed in 10% NBF for 24 h, dehydrated, and paraffin–embedded. Coronal sections were cut at 5 μm, with every tenth section mounted onto ~60 slides with 3 sections on each slide. This method allows to cover the entire brain and eliminate the likelihood of counting the same transcript twice. Sections were air-dried overnight at room temperature and stored at 4 °C until required.

Detection of mouse *Avp* and *Avpr* (*Avpr1a*) was performed separately on paraffin sections using Advanced Cell Diagnostics (ACD) RNAscope 2.5 LS Reagent Kit (#322100) and two RNAscope 2.5 LS probes, namely Mm-AVP-O1 (#472268) and Mm-AVPR1a (#418068). The kidney and prefrontal cortex served as positive and negative controls for AVPR1a, respectively. As with AVP, magnocellular cells of the PVH and SON served as positive controls while the brain from AVP–knockout mouse served a negative control.

Slides were baked at 60 °C for 1 h, deparaffinized, incubated with hydrogen peroxide for 10 min at room temperature, pretreated with Target Retrieval Reagent (#322001) for 20 min at 100 °C and with Protease III for 30 min at 40 °C. Probe hybridization and signal amplification were performed as per the manufacturer’s instructions for chromogenic assays.

Following RNAscope assay, the slides were scanned at ×20 magnification and the digital image analysis was successfully validated using the CaseViewer 2.4 (3DHISTECH) software. The same software was employed to capture and prepare images for the figures in the article. Images of control tissues were taken using microscope Leica DM 1000 LED. Detection of *Avp*- and *Avpr1a*-positive cells was also performed using the QuPath-0.2.3 (University of Edinburgh, UK) software based on receptor intensity thresholds, size, and shape.

### Quantitation, Validation and Statistical Analysis

Data were analyzed by Student’s *t*-test and one-way analysis of variance (ANOVA) followed by Tukey’s multiple comparisons tests using GraphPad Prism 10.2.2 version (La Jolla, CA). Significance was set at *P* < 0.05. For simplicity and clarity, exact test results and exact *P* values are not presented.

## Supplementary Material

Supplement 1

## References

[1] Brownstein M. J., Russell J. T., Gainer H., Synthesis, transport, and release of posterior pituitary hormones. Science 207, 373–378 (1980). 6153132 10.1126/science.6153132

[2] Young W. S. 3rd, Gainer H., Transgenesis and the study of expression, cellular targeting and function of oxytocin, vasopressin and their receptors. Neuroendocrinology 78, 185–203 (2003). 14583651 10.1159/000073702

[3] Silva Y. J., Moffat R. C., Walt A. J., Vasopressin effect on portal and systemic hemodynamics. Studies in intact, unanesthetized humans. JAMA 210, 1065–1068 (1969). 5394424

[4] Stockand J. D. , Chronic activation of vasopressin-2 receptors induces hypertension in Liddle mice by promoting Na(+) and water retention. Am J Physiol Renal Physiol 323, F468–F478 (2022). 35900342 10.1152/ajprenal.00384.2021 PMC9485005

[5] Lukas M., Neumann I. D., Oxytocin and vasopressin in rodent behaviors related to social dysfunctions in autism spectrum disorders. Behav Brain Res 251, 85–94 (2013). 22981649 10.1016/j.bbr.2012.08.011

[6] Lukas M., Toth I., Veenema A. H., Neumann I. D., Oxytocin mediates rodent social memory within the lateral septum and the medial amygdala depending on the relevance of the social stimulus: male juvenile versus female adult conspecifics. Psychoneuroendocrinology 38, 916–926 (2013). 23102690 10.1016/j.psyneuen.2012.09.018

[7] Veenema A. H., Neumann I. D., Central vasopressin and oxytocin release: regulation of complex social behaviours. Prog Brain Res 170, 261–276 (2008). 18655888 10.1016/S0079-6123(08)00422-6

[8] Meyer-Lindenberg A., Domes G., Kirsch P., Heinrichs M., Oxytocin and vasopressin in the human brain: social neuropeptides for translational medicine. Nat Rev Neurosci 12, 524–538 (2011). 21852800 10.1038/nrn3044

[9] Albers H. E., Species, sex and individual differences in the vasotocin/vasopressin system: relationship to neurochemical signaling in the social behavior neural network. Front Neuroendocrinol 36, 49–71 (2015). 25102443 10.1016/j.yfrne.2014.07.001 PMC4317378

[10] Dumais K. M., Veenema A. H., Vasopressin and oxytocin receptor systems in the brain: Sex differences and sex-specific regulation of social behavior. Front Neuroendocrinol 40, 1–23 (2016). 25951955 10.1016/j.yfrne.2015.04.003 PMC4633405

[11] French J. A., Taylor J. H., Mustoe A. C., Cavanaugh J., Neuropeptide diversity and the regulation of social behavior in New World primates. Front Neuroendocrinol 42, 18–39 (2016). 27020799 10.1016/j.yfrne.2016.03.004 PMC5030117

[12] Phelps S. M., Okhovat M., Berrio A., Individual Differences in Social Behavior and Cortical Vasopressin Receptor: Genetics, Epigenetics, and Evolution. Frontiers in neuroscience 11, 537 (2017). 29085274 10.3389/fnins.2017.00537 PMC5649215

[13] Lim M. M. , Enhanced partner preference in a promiscuous species by manipulating the expression of a single gene. Nature 429, 754–757 (2004). 15201909 10.1038/nature02539

[14] Fink S., Excoffier L., Heckel G., High variability and non-neutral evolution of the mammalian avpr1a gene. BMC Evol Biol 7, 176 (2007). 17900345 10.1186/1471-2148-7-176 PMC2121647

[15] Ren D., Chin K. R., French J. A., Molecular variation in AVP and AVPR1a in New World monkeys (Primates, Platyrrhini): evolution and implications for social monogamy. PloS one 9, e111638 (2014). 25360668 10.1371/journal.pone.0111638 PMC4216101

[16] Bielsky I. F., Hu S. B., Szegda K. L., Westphal H., Young L. J., Profound impairment in social recognition and reduction in anxiety-like behavior in vasopressin V1a receptor knockout mice. Neuropsychopharmacology 29, 483–493 (2004). 14647484 10.1038/sj.npp.1300360

[17] Neumann I. D., Landgraf R., Balance of brain oxytocin and vasopressin: implications for anxiety, depression, and social behaviors. Trends Neurosci 35, 649–659 (2012). 22974560 10.1016/j.tins.2012.08.004

[18] Lago T. R. , The novel vasopressin receptor (Avpr1a) antagonist SRX246 reduces anxiety in an experimental model in humans: a randomized proof-of-concept study. Psychopharmacology (Berl) 238, 2393–2403 (2021). 33970290 10.1007/s00213-021-05861-4 PMC8376758

[19] Thompson R. R., George K., Walton J. C., Orr S. P., Benson J., Sex-specific influences of vasopressin on human social communication. Proc Natl Acad Sci U S A 103, 7889–7894 (2006). 16682649 10.1073/pnas.0600406103 PMC1472540

[20] Bielsky I. F., Hu S. B., Ren X., Terwilliger E. F., Young L. J., The V1a vasopressin receptor is necessary and sufficient for normal social recognition: a gene replacement study. Neuron 47, 503–513 (2005). 16102534 10.1016/j.neuron.2005.06.031

[21] Veenema A. H., Bredewold R., De Vries G. J., Vasopressin regulates social recognition in juvenile and adult rats of both sexes, but in sex- and age-specific ways. Horm Behav 61, 50–56 (2012). 22033278 10.1016/j.yhbeh.2011.10.002 PMC3264802

[22] Winslow J. T., Hastings N., Carter C. S., Harbaugh C. R., Insel T. R., A role for central vasopressin in pair bonding in monogamous prairie voles. Nature 365, 545–548 (1993). 8413608 10.1038/365545a0

[23] Insel T. R., Hulihan T. J., A sex-specific mechanism for pair bonding: oxytocin and partner preference formation in monogamous voles. Behav Neurosci 109, 782–789 (1995). 7576222 10.1037//0735-7044.109.4.782

[24] Insel T. R., Wang Z. X., Ferris C. F., Patterns of brain vasopressin receptor distribution associated with social organization in microtine rodents. J Neurosci 14, 5381–5392 (1994). 8083743 10.1523/JNEUROSCI.14-09-05381.1994 PMC6577077

[25] Wang Z., Young L. J., Liu Y., Insel T. R., Species differences in vasopressin receptor binding are evident early in development: comparative anatomic studies in prairie and montane voles. J Comp Neurol 378, 535–546 (1997). 9034909

[26] Lim M. M., Young L. J., Vasopressin-dependent neural circuits underlying pair bond formation in the monogamous prairie vole. Neuroscience 125, 35–45 (2004). 15051143 10.1016/j.neuroscience.2003.12.008

[27] Summy-Long J. Y., Kadekaro M., Role of circumventricular organs (CVO) in neuroendocrine responses: interactions of CVO and the magnocellular neuroendocrine system in different reproductive states. Clin Exp Pharmacol Physiol 28, 590–601 (2001). 11458887 10.1046/j.1440-1681.2001.03491.x

[28] Stricker E. M., Huang W., Sved A. F., Early osmoregulatory signals in the control of water intake and neurohypophyseal hormone secretion. Physiol Behav 76, 415–421 (2002). 12117578 10.1016/s0031-9384(02)00752-7

[29] Ludwig M., Callahan M. F., Neumann I., Landgraf R., Morris M., Systemic osmotic stimulation increases vasopressin and oxytocin release within the supraoptic nucleus. J Neuroendocrinol 6, 369–373 (1994). 7987366 10.1111/j.1365-2826.1994.tb00595.x

[30] Ludwig M., Leng G., Autoinhibition of supraoptic nucleus vasopressin neurons in vivo: a combined retrodialysis/electrophysiological study in rats. The European journal of neuroscience 9, 2532–2540 (1997). 9517458 10.1111/j.1460-9568.1997.tb01682.x

[31] Wang B. C., Share L., Crofton J. T., Central infusion of vasopressin decreased plasma vasopressin concentration in dogs. Am J Physiol 243, E365–369 (1982). 7137341 10.1152/ajpendo.1982.243.5.E365

[32] Wotjak C. T., Ludwig M., Landgraf R., Vasopressin facilitates its own release within the rat supraoptic nucleus in vivo. Neuroreport 5, 1181–1184 (1994). 7919160 10.1097/00001756-199406020-00005

[33] Zaidi M. , Actions of pituitary hormones beyond traditional targets. J Endocrinol 237, R83–R98 (2018). 29555849 10.1530/JOE-17-0680 PMC5924585

[34] Abe E. , TSH is a negative regulator of skeletal remodeling. Cell 115, 151–162 (2003). 14567913 10.1016/s0092-8674(03)00771-2

[35] Tamma R. , Regulation of bone remodeling by vasopressin explains the bone loss in hyponatremia. Proc Natl Acad Sci U S A 110, 18644–18649 (2013). 24167258 10.1073/pnas.1318257110 PMC3831977

[36] Sun L. , Oxytocin regulates body composition. Proc Natl Acad Sci U S A 10.1073/pnas.1913611116 (2019). PMC6936484 31843930

[37] Sun L. , Functions of vasopressin and oxytocin in bone mass regulation. Proc Natl Acad Sci U S A 113, 164–169 (2016). 26699482 10.1073/pnas.1523762113 PMC4711832

[38] Tamma R. , Oxytocin is an anabolic bone hormone. Proc Natl Acad Sci U S A 106, 7149–7154 (2009). 19369205 10.1073/pnas.0901890106 PMC2678458

[39] Phillips P. A. , Localization of vasopressin binding sites in rat tissues using specific V1 and V2 selective ligands. Endocrinology 126, 1478–1484 (1990). 2307115 10.1210/endo-126-3-1478

[40] Paxinos G., Franklin K. B. J., The Mouse Brain in Stereotaxic Coordinates (Academic Press, New York, ed. 3rd, 2007).

[41] DeVries G. J., Buijs R. M., Swaab D. F., Ontogeny of the vasopressinergic neurons of the suprachiasmatic nucleus and their extra-hypothalamic projections in the rat brain-presence of a sex difference in the lateral septum. Brain Research 218, 67–78 (1981). 7023607 10.1016/0006-8993(81)90989-6

[42] De Vries G. J., Buijs R. M., The origin of the vasopressinergic and oxytocinergic innervation of the rat brain with special reference to the lateral septum. Brain Res 273, 307–317 (1983). 6311351 10.1016/0006-8993(83)90855-7

[43] Caffe A. R., Van Ryen P. C., Van der Woude T. P., Van Leeuwen F. W., Vasopressin and oxytocin systems in the brain and upper spinal cord of Macaca fascicularis. J Comp Neurol 287, 302–325 (1989). 2778107 10.1002/cne.902870304

[44] de Vries G. J., Buijs R. M., Sluiter A. A., Gonadal hormone actions on the morphology of the vasopressinergic innervation of the adult rat brain. Brain Res 298, 141–145 (1984). 6722551 10.1016/0006-8993(84)91157-0

[45] Miller M. A., DeVries G. J., al-Shamma H. A., Dorsa D. M., Decline of vasopressin immunoreactivity and mRNA levels in the bed nucleus of the stria terminalis following castration. J Neurosci 12, 2881–2887 (1992). 1494938 10.1523/JNEUROSCI.12-08-02881.1992 PMC6575668

[46] De Vries G. J., al-Shamma H. A., Sex differences in hormonal responses of vasopressin pathways in the rat brain. J Neurobiol 21, 686–693 (1990). 2394985 10.1002/neu.480210503

[47] De Vries G. J., Wang Z., Bullock N. A., Numan S., Sex differences in the effects of testosterone and its metabolites on vasopressin messenger RNA levels in the bed nucleus of the stria terminalis of rats. J Neurosci 14, 1789–1794 (1994). 8126571 10.1523/JNEUROSCI.14-03-01789.1994 PMC6577591

[48] Joca L., Zuloaga D. G., Raber J., Siegel J. A., Long-term effects of early adolescent methamphetamine exposure on depression-like behavior and the hypothalamic vasopressin system in mice. Dev Neurosci 36, 108–118 (2014). 24686407 10.1159/000360001 PMC5921901

[49] Steinman M. Q. , Hypothalamic vasopressin systems are more sensitive to the long term effects of social defeat in males versus females. Psychoneuroendocrinology 51, 122–134 (2015). 25306217 10.1016/j.psyneuen.2014.09.009 PMC4268083

[50] Wang Z., Species differences in the vasopressin-immunoreactive pathways in the bed nucleus of the stria terminalis and medial amygdaloid nucleus in prairie voles (Microtus ochrogaster) and meadow voles (Microtus pennsylvanicus). Behav Neurosci 109, 305–311 (1995). 7619320 10.1037//0735-7044.109.2.305

[51] Wang Z., Zhou L., Hulihan T. J., Insel T. R., Immunoreactivity of central vasopressin and oxytocin pathways in microtine rodents: a quantitative comparative study. J Comp Neurol 366, 726–737 (1996). 8833119 10.1002/(SICI)1096-9861(19960318)366:4<726::AID-CNE11>3.0.CO;2-D

[52] Wang Y., Xu L., Pan Y., Wang Z., Zhang Z., Species differences in the immunoreactive expression of oxytocin, vasopressin, tyrosine hydroxylase and estrogen receptor alpha in the brain of Mongolian gerbils (Meriones unguiculatus) and Chinese striped hamsters (Cricetulus barabensis). PloS one 8, e65807 (2013). 23762431 10.1371/journal.pone.0065807 PMC3676338

[53] Ostrowski N. L. , Distribution of V1a and V2 vasopressin receptor messenger ribonucleic acids in rat liver, kidney, pituitary and brain. Endocrinology 131, 533–535 (1992). 1535312 10.1210/endo.131.1.1535312

[54] Zhang C. L. , Arcuate NPY is involved in salt-induced hypertension via modulation of paraventricular vasopressin and brain-derived neurotrophic factor. J Cell Physiol 237, 2574–2588 (2022). 35312067 10.1002/jcp.30719 PMC9544553

[55] Pineda R., Sabatier N., Ludwig M., Millar R. P., Leng G., A Direct Neurokinin B Projection from the Arcuate Nucleus Regulates Magnocellular Vasopressin Cells of the Supraoptic Nucleus. J Neuroendocrinol 28 (2016). 10.1111/jne.12342 26610724

[56] Maejima Y. , Oxytocinergic circuit from paraventricular and supraoptic nuclei to arcuate POMC neurons in hypothalamus. FEBS Lett 588, 4404–4412 (2014). 25448678 10.1016/j.febslet.2014.10.010

[57] Meyer A. H., Langhans W., Scharrer E., Vasopressin reduces food intake in goats. Q J Exp Physiol 74, 465–473 (1989). 2798756 10.1113/expphysiol.1989.sp003294

[58] Langhans W., Delprete E., Scharrer E., Mechanisms of vasopressin’s anorectic effect. Physiol Behav 49, 169–176 (1991). 1826789 10.1016/0031-9384(91)90251-i

[59] Kalsbeek A., Fliers E., Hofman M. A., Swaab D. F., Buijs R. M., Vasopressin and the output of the hypothalamic biological clock. J Neuroendocrinol 22, 362–372 (2010). 20088910 10.1111/j.1365-2826.2010.01956.x

[60] Trudel E., Bourque C. W., Central clock excites vasopressin neurons by waking osmosensory afferents during late sleep. Nat Neurosci 13, 467–474 (2010). 20190744 10.1038/nn.2503

[61] Gizowski C., Zaelzer C., Bourque C. W., Clock-driven vasopressin neurotransmission mediates anticipatory thirst prior to sleep. Nature 537, 685–688 (2016). 27680940 10.1038/nature19756

[62] Dai J., Swaab D. F., Buijs R. M., Recovery of axonal transport in “dead neurons”. Lancet 351, 499–500 (1998). 10.1016/S0140-6736(05)78689-X 9482451

[63] Dai J., Swaab D. F., Van der Vliet J., Buijs R. M., Postmortem tracing reveals the organization of hypothalamic projections of the suprachiasmatic nucleus in the human brain. J Comp Neurol 400, 87–102 (1998). 9762868 10.1002/(sici)1096-9861(19981012)400:1<87::aid-cne6>3.0.co;2-p

[64] Rohr K. E., Telega A., Savaglio A., Evans J. A., Vasopressin regulates daily rhythms and circadian clock circuits in a manner influenced by sex. Horm Behav 127, 104888 (2021). 33202247 10.1016/j.yhbeh.2020.104888 PMC7855892

[65] Yoshimura M., Conway-Campbell B., Ueta Y., Arginine vasopressin: Direct and indirect action on metabolism. Peptides 142, 170555 (2021). 33905792 10.1016/j.peptides.2021.170555 PMC8270887

[66] Young L. J., Toloczko D., Insel T. R., Localization of vasopressin (V1a) receptor binding and mRNA in the rhesus monkey brain. J Neuroendocrinol 11, 291–297 (1999). 10223283 10.1046/j.1365-2826.1999.00332.x

[67] Szczepanska-Sadowska E., Wsol A., Cudnoch-Jedrzejewska A., Zera T., Complementary Role of Oxytocin and Vasopressin in Cardiovascular Regulation. International journal of molecular sciences 22 (2021). 10.3390/ijms222111465 PMC8584236 34768894

[68] Edwards I. J., Deuchars S. A., Deuchars J., The intermedius nucleus of the medulla: a potential site for the integration of cervical information and the generation of autonomic responses. J Chem Neuroanat 38, 166–175 (2009). 19790285 10.1016/j.jchemneu.2009.01.001

[69] Zanutto B. S., Valentinuzzi M. E., Segura E. T., Neural set point for the control of arterial pressure: role of the nucleus tractus solitarius. Biomed Eng Online 9, 4 (2010). 20064256 10.1186/1475-925X-9-4 PMC3224897

[70] Baumeister R. F., Leary M. R., The need to belong: desire for interpersonal attachments as a fundamental human motivation. Psychol Bull 117, 497–529 (1995). 7777651

[71] Wagner U. , Beautiful friendship: Social sharing of emotions improves subjective feelings and activates the neural reward circuitry. Soc Cogn Affect Neurosci 10, 801–808 (2015). 25298009 10.1093/scan/nsu121 PMC4448023

[72] Rigney N., Beaumont R., Petrulis A., Sex differences in vasopressin 1a receptor regulation of social communication within the lateral habenula and dorsal raphe of mice. Horm Behav 121, 104715 (2020). 32067962 10.1016/j.yhbeh.2020.104715 PMC7249673

[73] Rood B. D. , Site of origin of and sex differences in the vasopressin innervation of the mouse (Mus musculus) brain. J Comp Neurol 521, 2321–2358 (2013). 23239101 10.1002/cne.23288

[74] Tobin V. A. , An intrinsic vasopressin system in the olfactory bulb is involved in social recognition. Nature 464, 413–417 (2010). 20182426 10.1038/nature08826 PMC2842245

[75] Levy F., Kendrick K. M., Goode J. A., Guevara-Guzman R., Keverne E. B., Oxytocin and vasopressin release in the olfactory bulb of parturient ewes: changes with maternal experience and effects on acetylcholine, gamma-aminobutyric acid, glutamate and noradrenaline release. Brain Res 669, 197–206 (1995). 7712175 10.1016/0006-8993(94)01236-b

[76] Reed M. D., Price K. E., Archbold J., Moffa A., Febo M., Predator odor-evoked BOLD activation in the awake rat: modulation by oxytocin and V(1)a vasopressin receptor antagonists. Brain Res 1494, 70–83 (2013). 23219972 10.1016/j.brainres.2012.11.045 PMC3809104

[77] De Wied D., Long term effect of vasopressin on the maintenance of a conditioned avoidance response in rats. Nature 232, 58–60 (1971). 4326313 10.1038/232058a0

[78] Zhang L. , Vasopressin induces depolarization and state-dependent firing patterns in rat thalamic paraventricular nucleus neurons in vitro. Am J Physiol Regul Integr Comp Physiol 290, R1226–1232 (2006). 16339383 10.1152/ajpregu.00770.2005

[79] Matsuguchi H., Sharabi F. M., Gordon F. J., Johnson A. K., Schmid P. G., Blood pressure and heart rate responses to microinjection of vasopressin into the nucleus tractus solitarius region of the rat. Neuropharmacology 21, 687–693 (1982). 7121740 10.1016/0028-3908(82)90012-0

[80] Faraci F. M., Mayhan W. G., Farrell W. J., Heistad D. D., Humoral regulation of blood flow to choroid plexus: role of arginine vasopressin. Circ Res 63, 373–379 (1988). 3396158 10.1161/01.res.63.2.373

