# Supplementary material for: Gender–specific Single Transcript Level Atlas of Vasopressin and its Receptor (AVPR1a) in the Mouse Brain
Source: bioRxiv. 2024 Dec 10:2024.12.09.627541. Preprint. [Version 1] doi: 10.1101/2024.12.09.627541 (PMC11661174; doi:10.1101/2024.12.09.627541)

AVP-positive cells in brain divisions

Supplementary Figure 1A

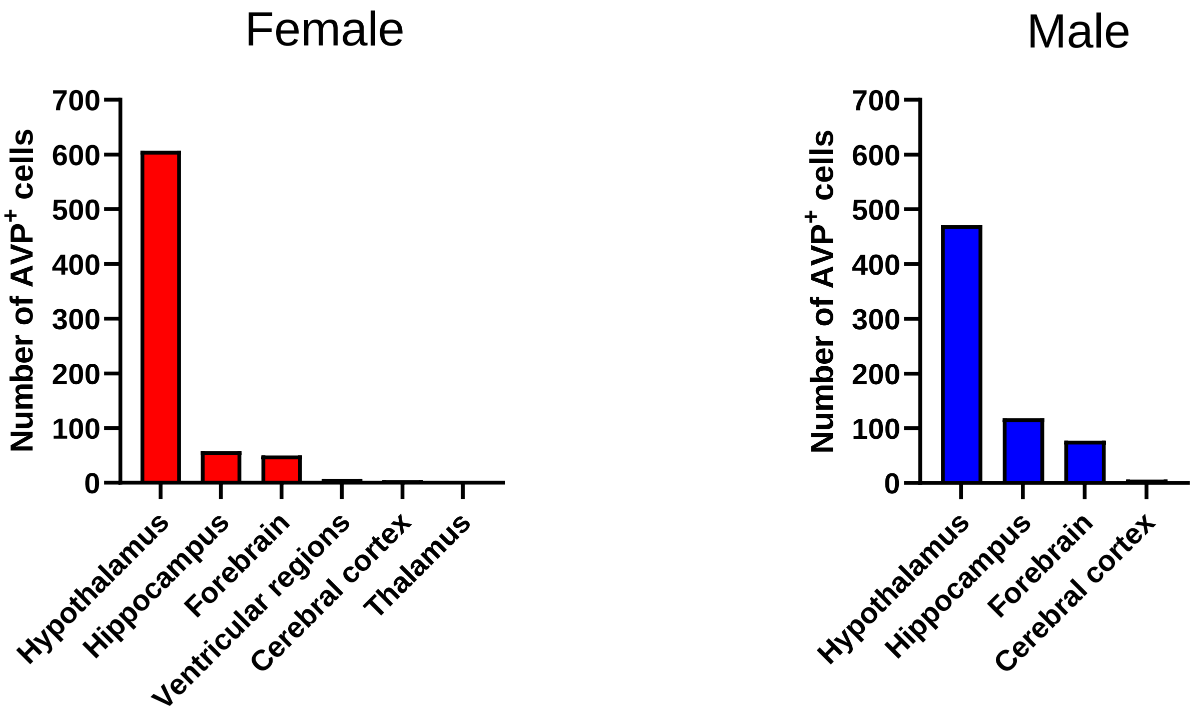

Supplementary Figure 1B

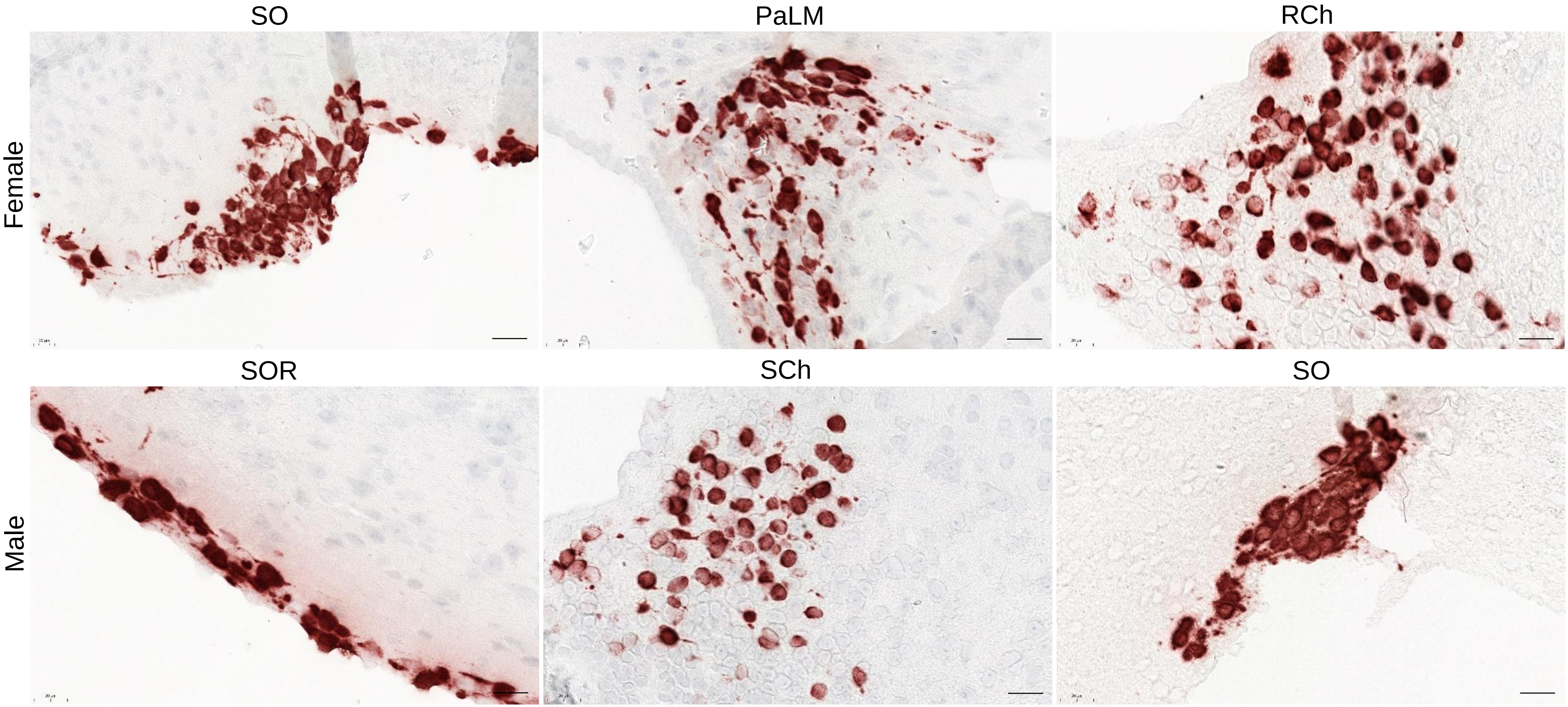

Supplementary Figure 1B

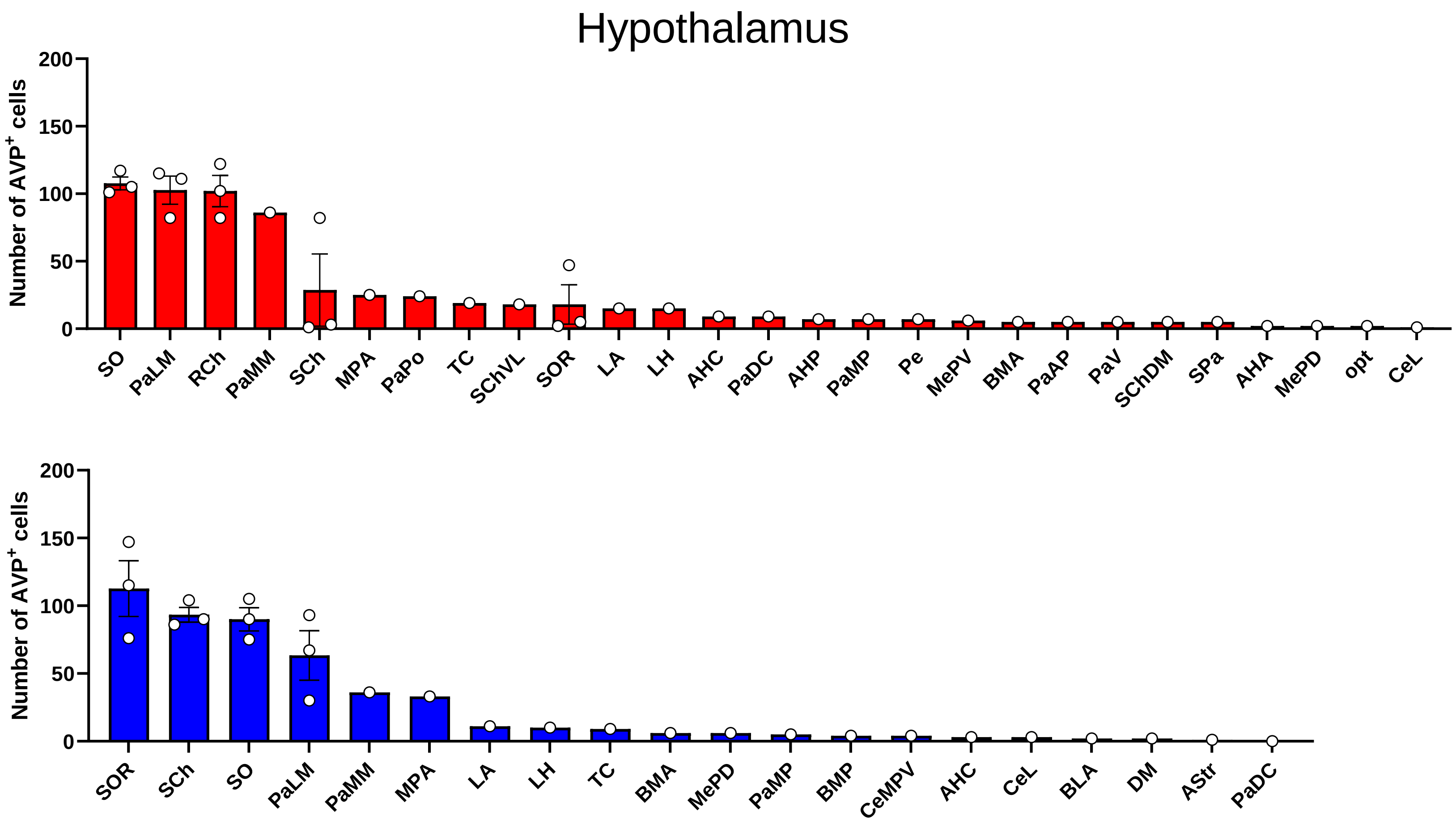

Supplementary Figure 1B

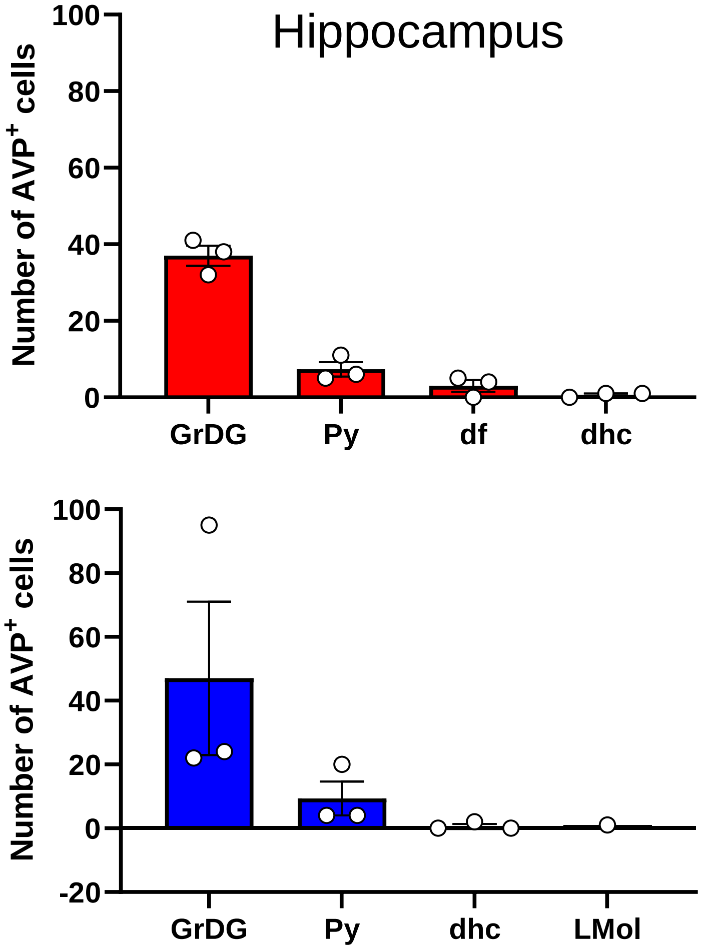

Supplementary Figure 1B

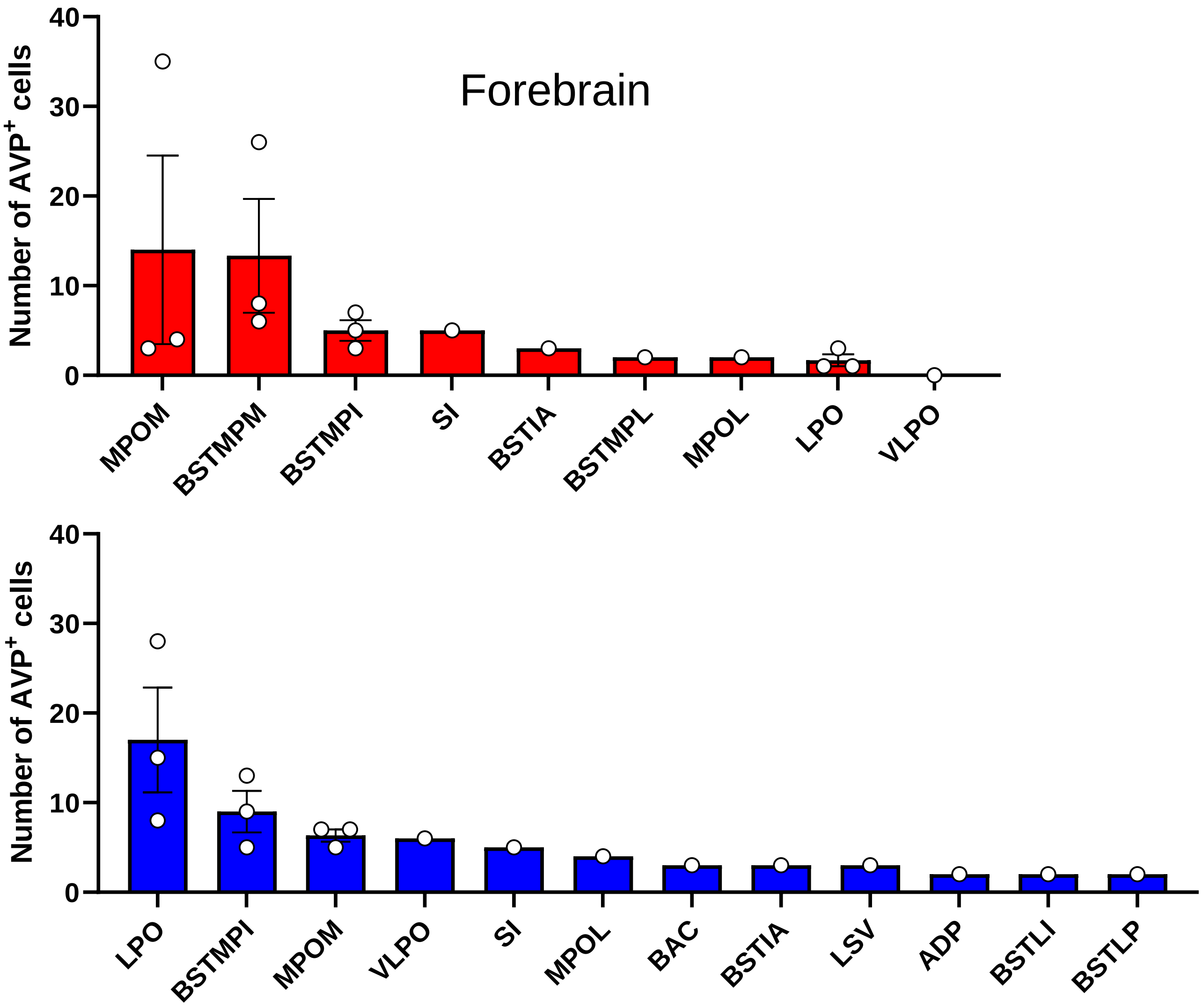

Supplementary Figure 1B

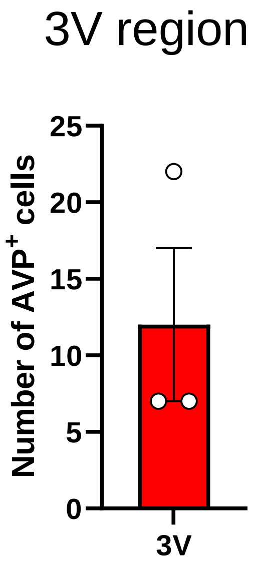

Supplementary Figure 1B

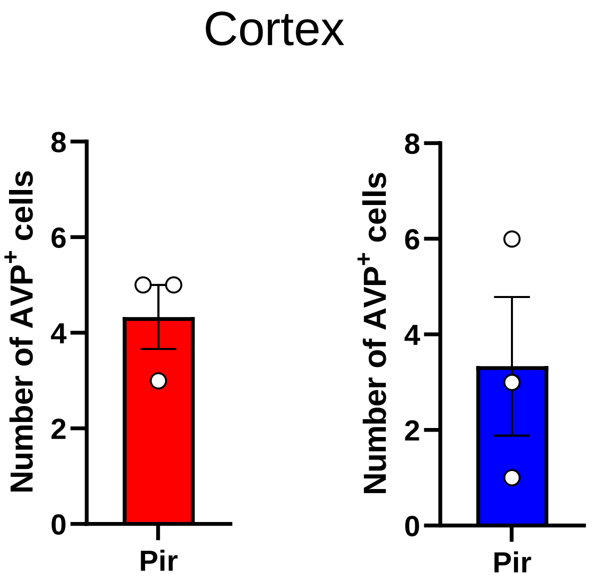

Supplementary Figure 1B

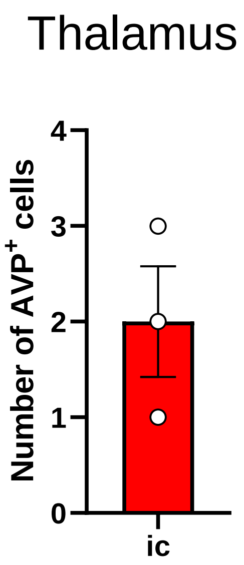

AVPR1a transcript number in brain divisions

Supplementary Figure 2A

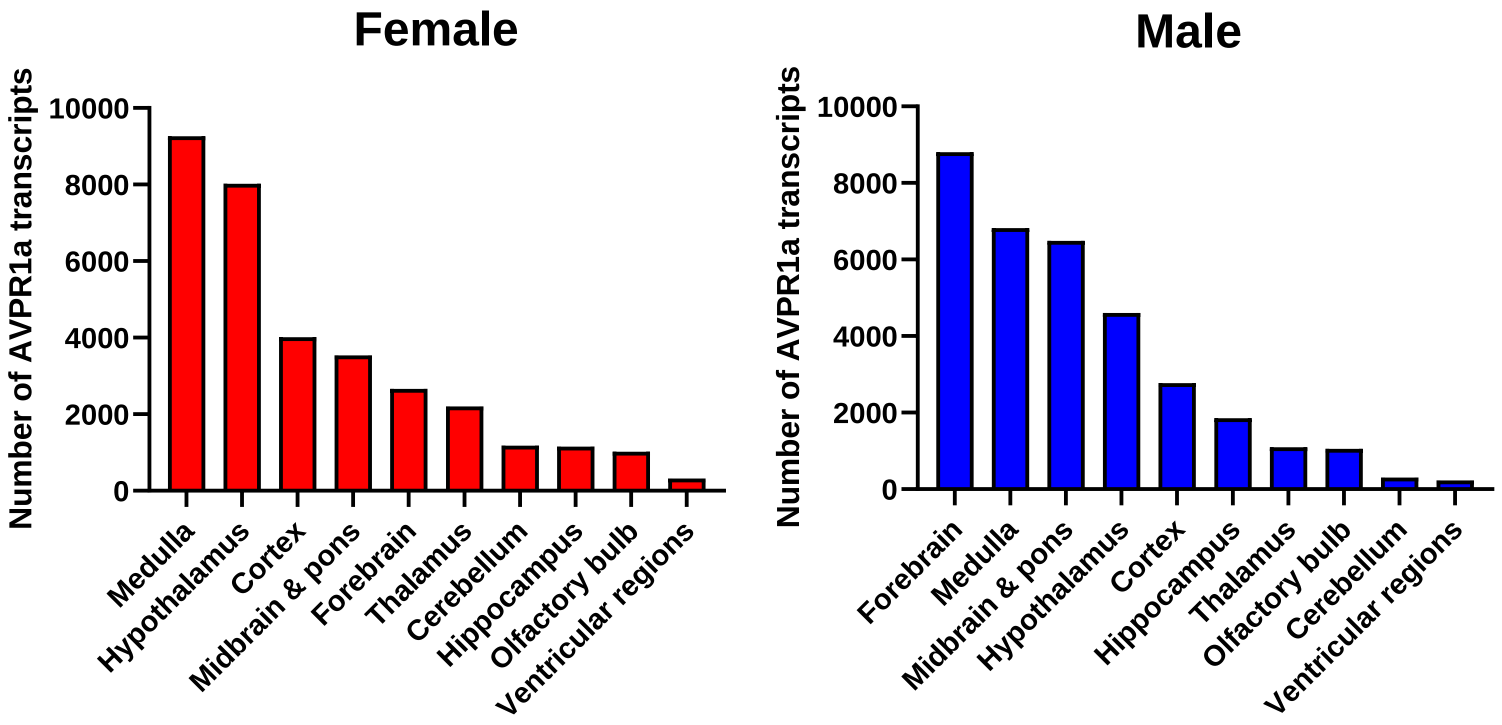

Supplementary Figure 2B

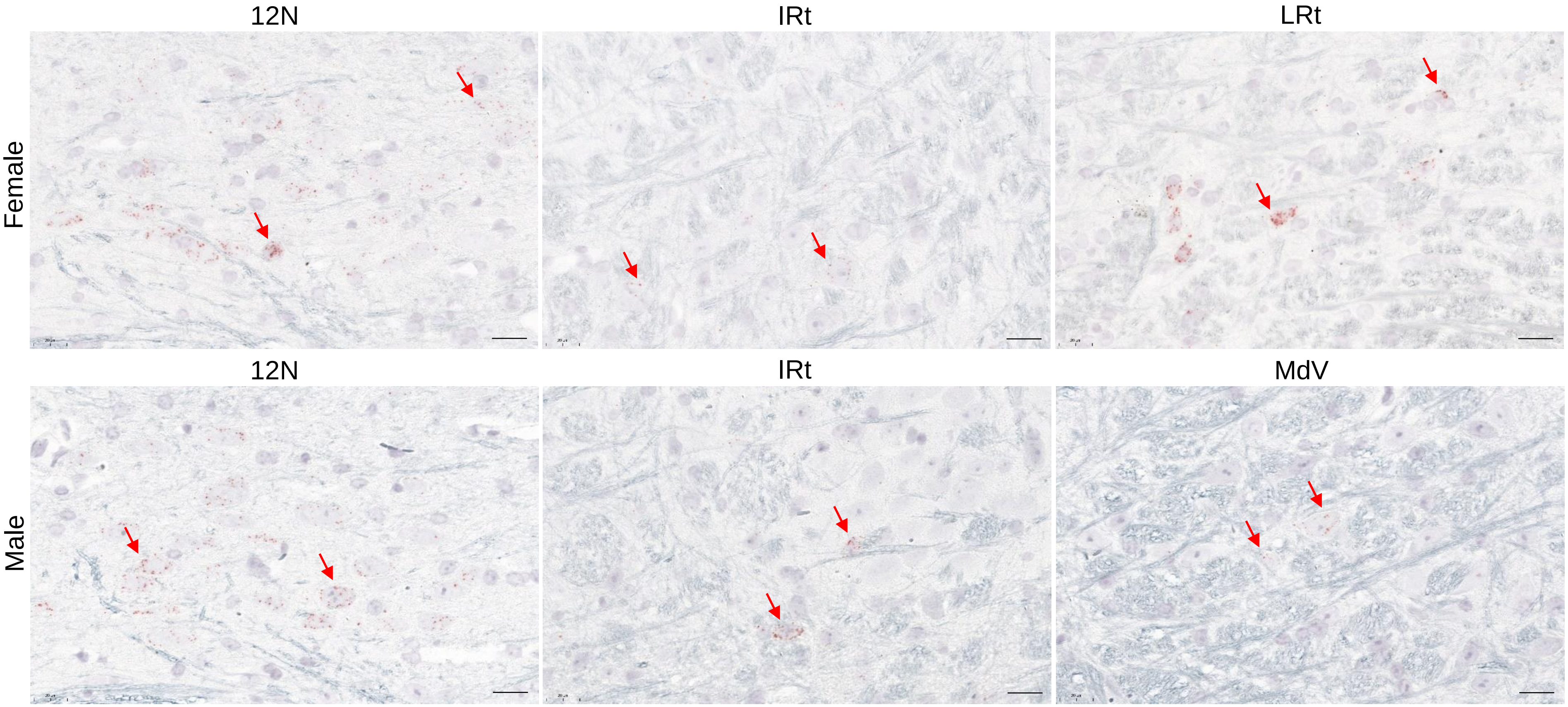

Supplementary Figure 2B

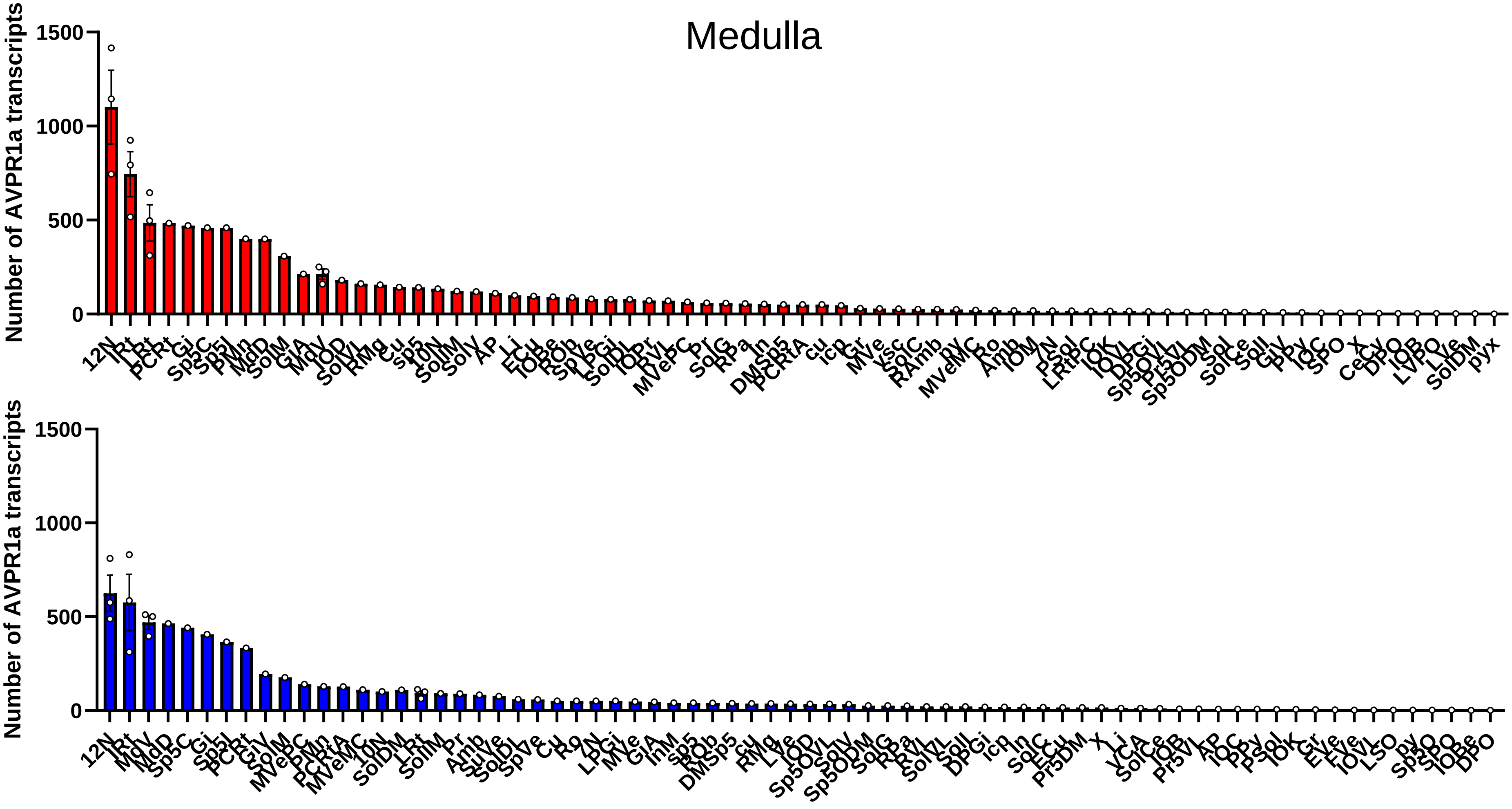

Supplementary Figure 2B

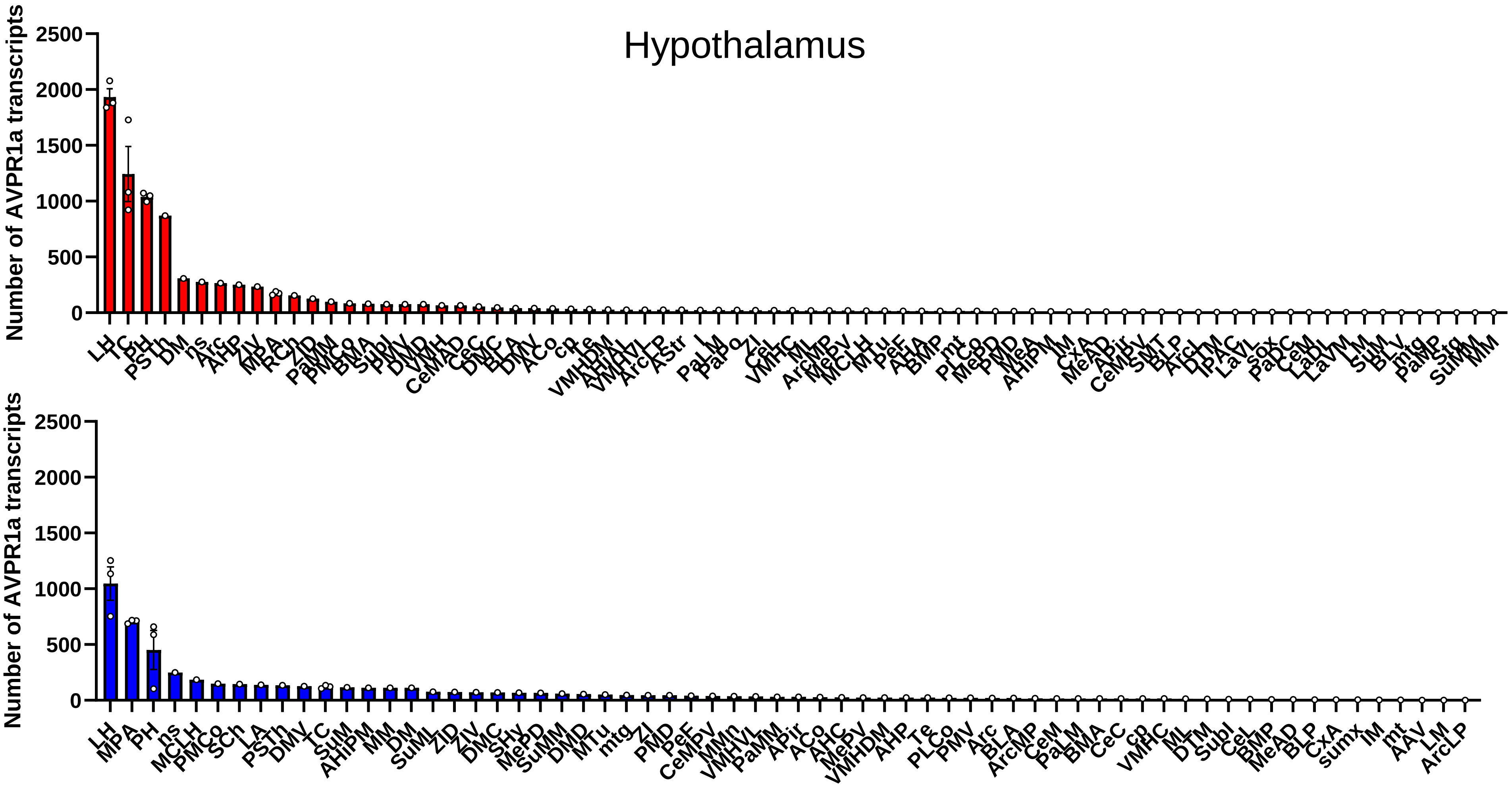

Supplementary Figure 2B

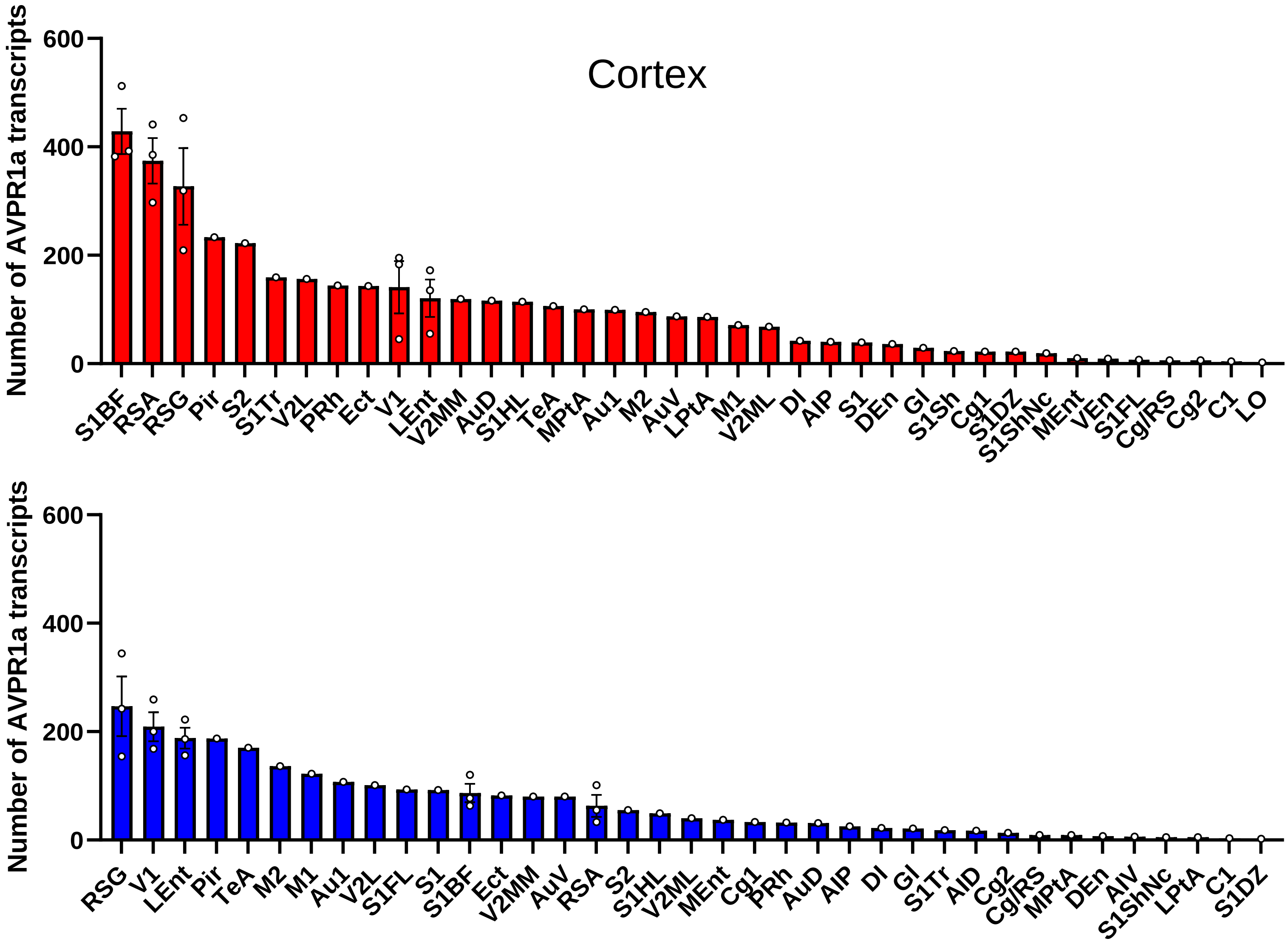

Supplementary Figure 2B

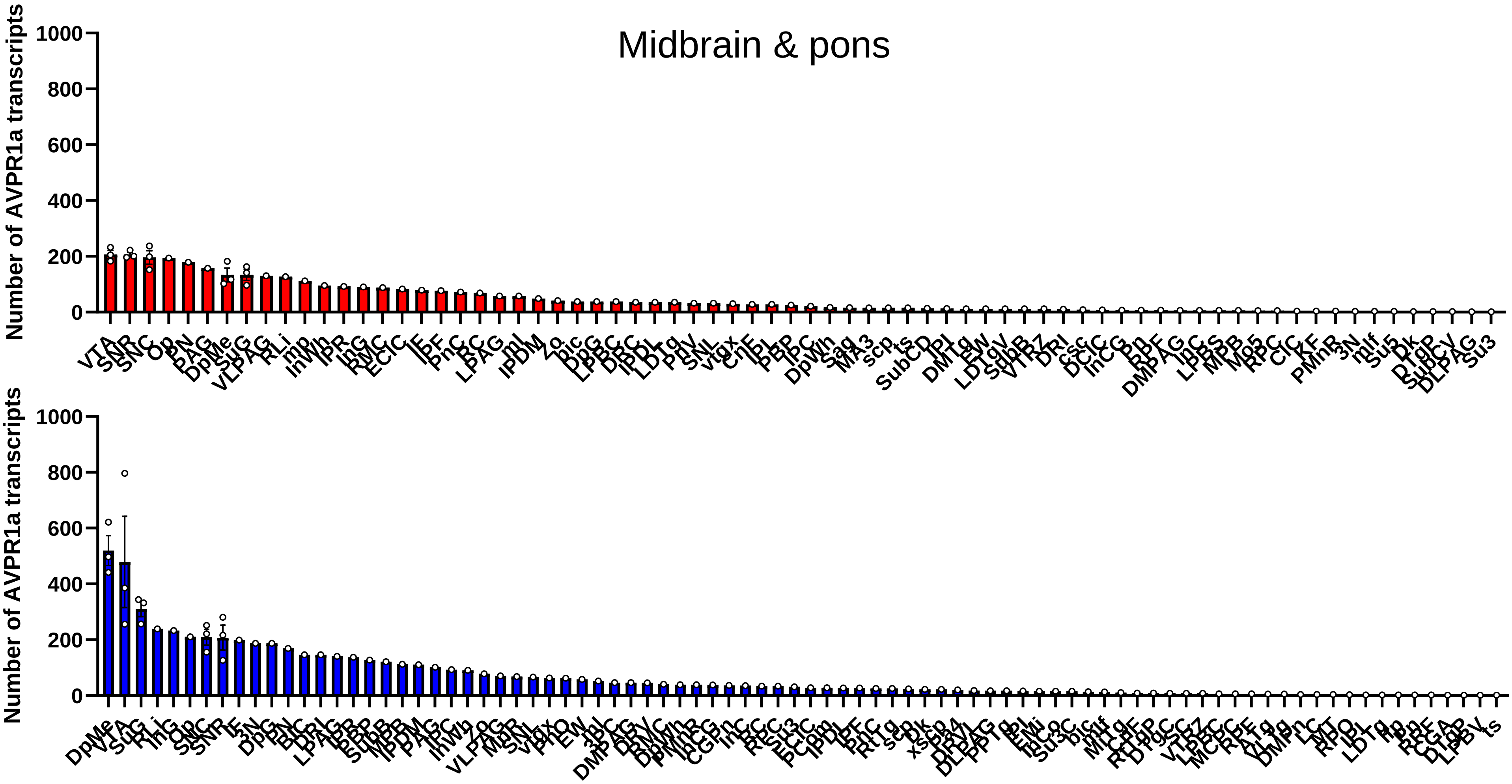

Supplementary Figure 2B

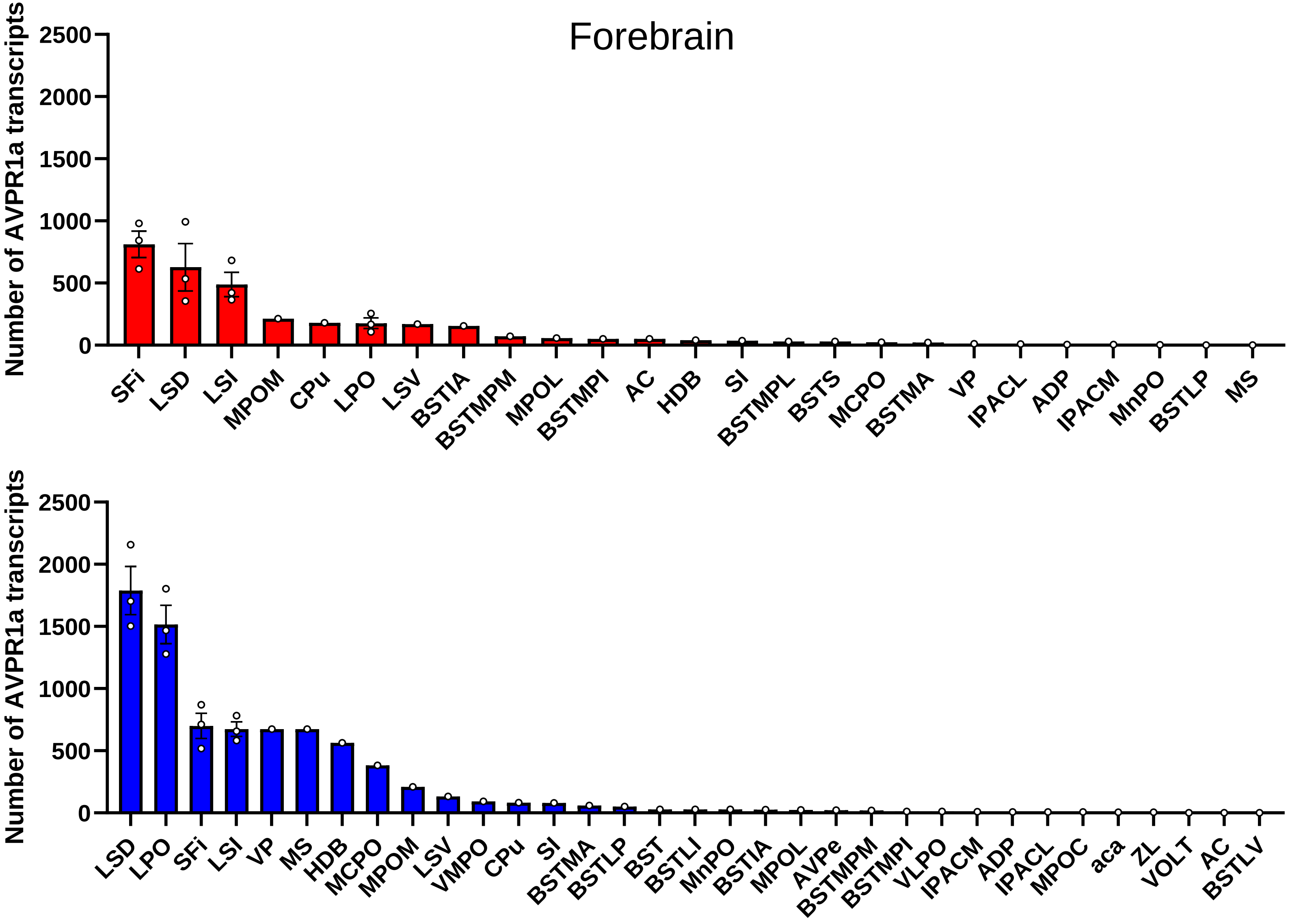

Supplementary Figure 2B

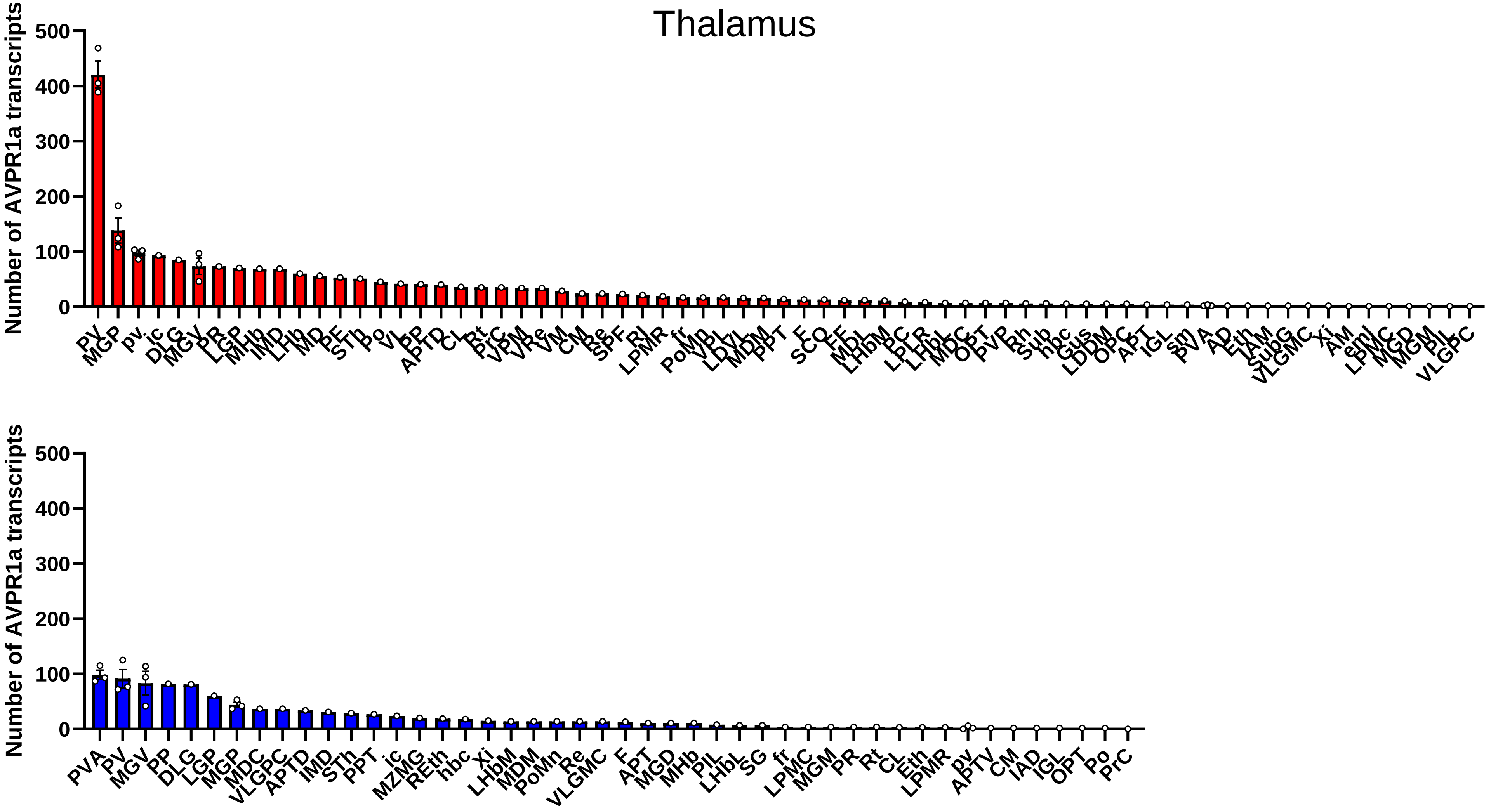

Supplementary Figure 2B

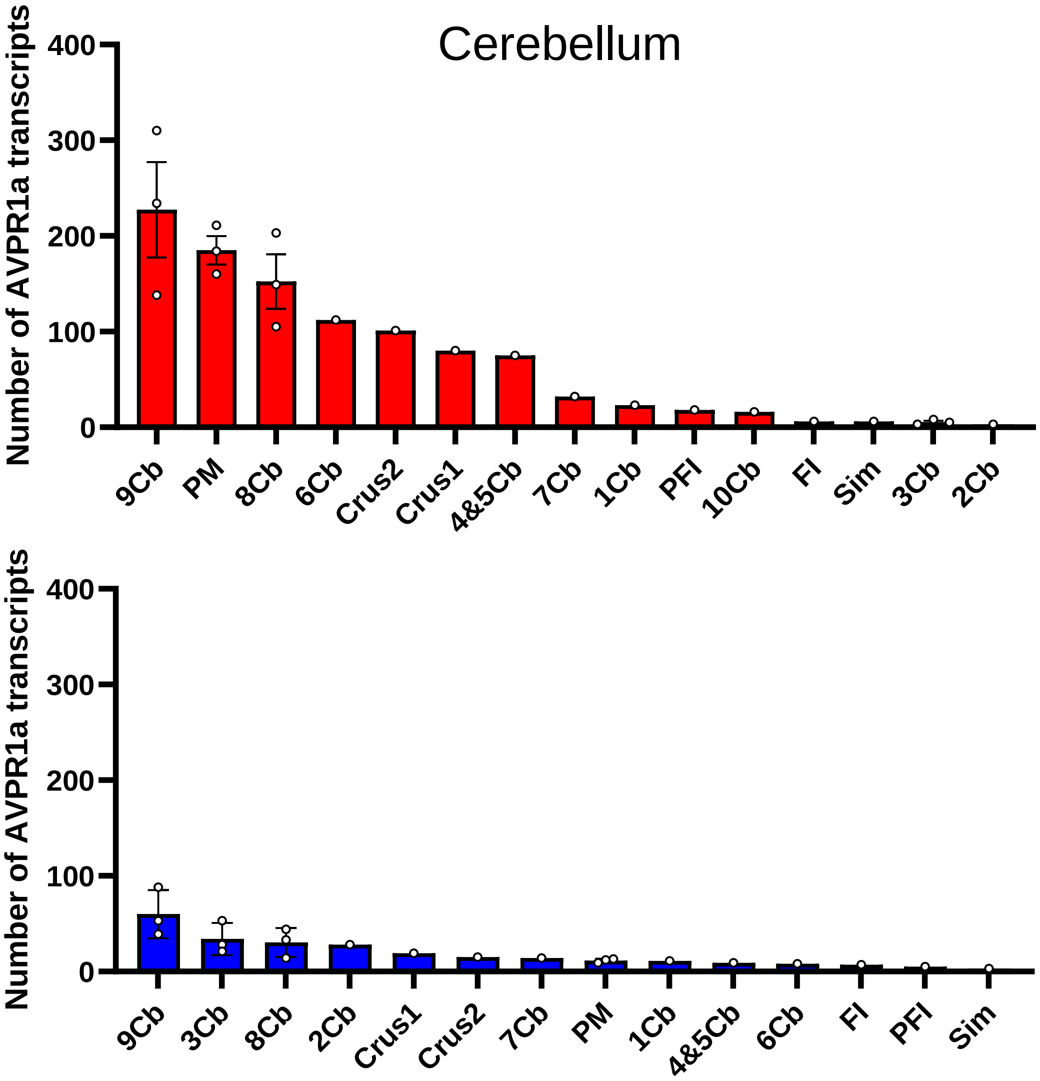

Supplementary Figure 2B

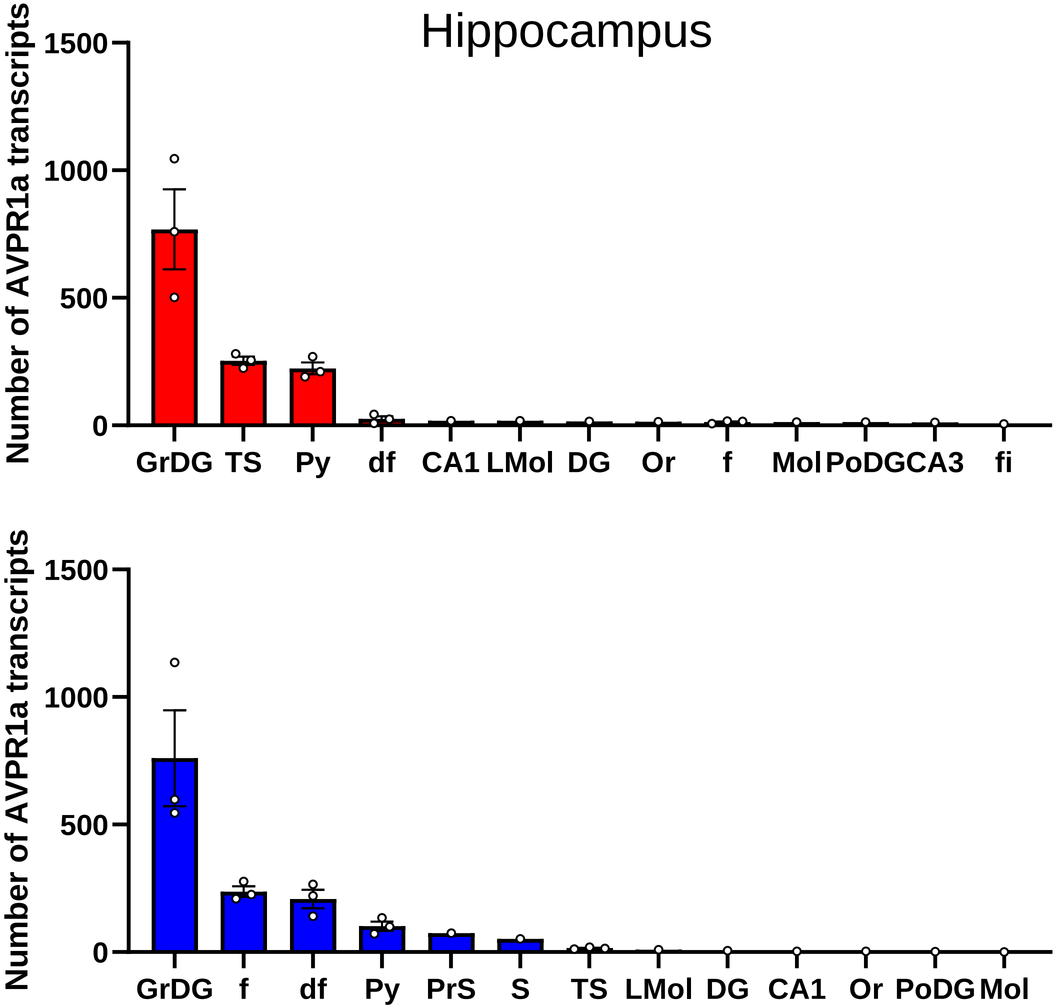

Supplementary Figure 2B

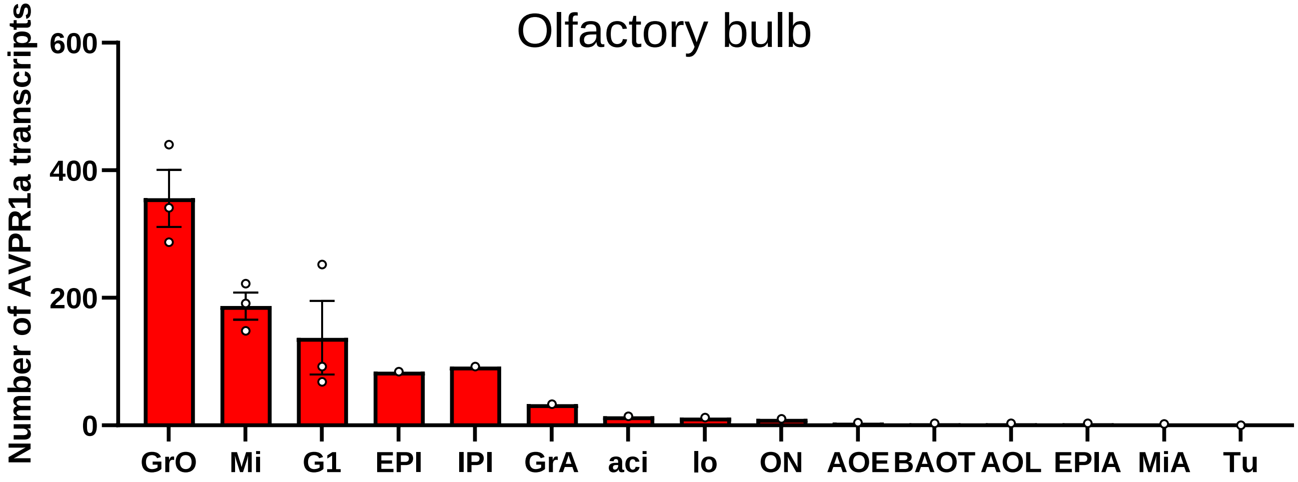

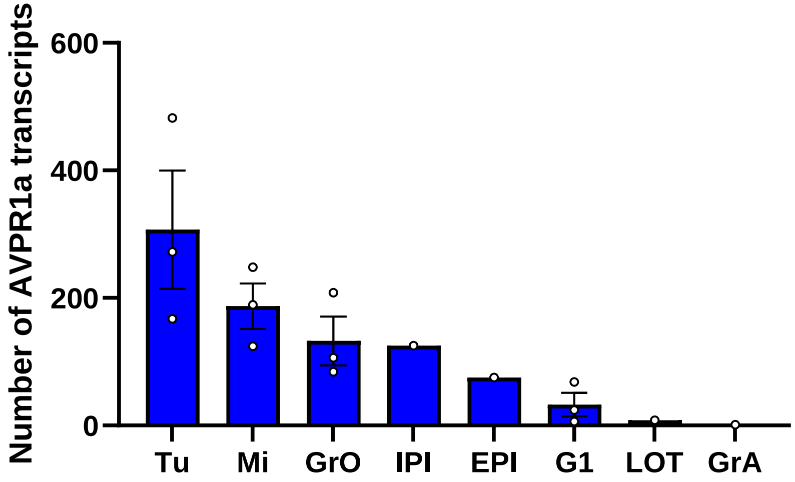

Supplementary Figure 2B

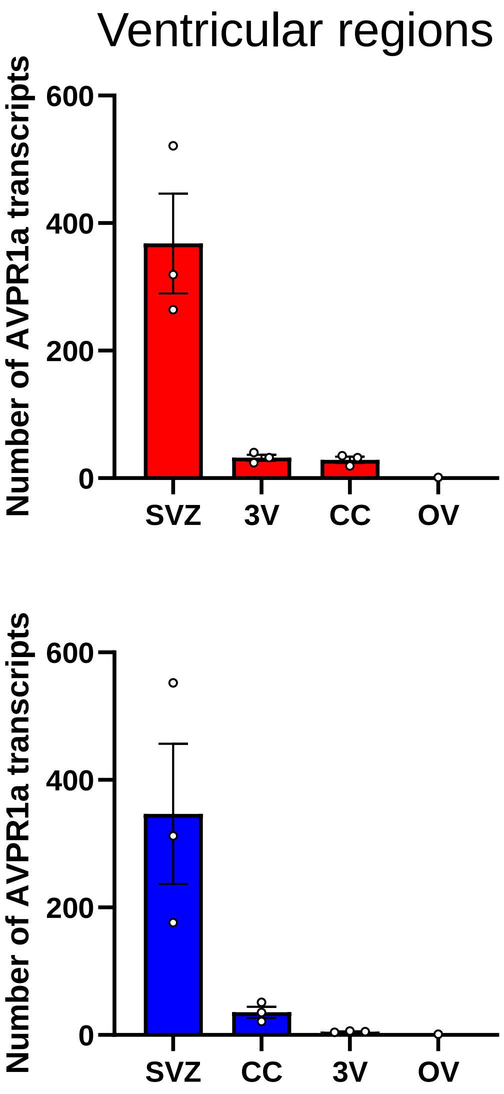

Supplement: Supplement 1 [file NIHPP2024.12.09.627541v1-supplement-1.pdf]
